# Improving CO_2_ photoconversion with ionic liquid and Co single atoms

**DOI:** 10.1038/s41467-023-36980-5

**Published:** 2023-03-16

**Authors:** Yang Liu, Jianhui Sun, Houhou Huang, Linlu Bai, Xiaomeng Zhao, Binhong Qu, Lunqiao Xiong, Fuquan Bai, Junwang Tang, Liqiang Jing

**Affiliations:** 1 Department Key Laboratory of Functional Inorganic Materials Chemistry (Ministry of Education), School of Chemistry and Materials Science, International Joint Research Center and Lab for Catalytic Technology, Heilongjiang University, Harbin, Heilongjiang 150080 P. R. China; 2 Department Key Laboratory of Functional Inorganic Materials Chemistry (Ministry of Education), International Joint Research Center for Catalytic Technology, School of Physics, Heilongjiang University, Harbin, 150080 P. R. China; 3 International Joint Research Laboratory of Nano-Micro Architecture Chemistry, Institute of Theoretical Chemistry and College of Chemistry, Jilin University Changchun, 130021 Changchun, P. R. China; 4 Department of Chemical Engineering, University College London, Torrington Place, London, WC1E 7JE UK

**Keywords:** Photocatalysis, Energy, Photocatalysis

## Abstract

Photocatalytic CO_2_ conversion promises an ideal route to store solar energy into chemical bonds. However, sluggish electron kinetics and unfavorable product selectivity remain unresolved challenges. Here, an ionic liquid, 1-ethyl-3-methylimidazolium tetrafluoroborate, and borate-anchored Co single atoms were separately loaded on ultrathin g-C_3_N_4_ nanosheets. The optimized nanocomposite photocatalyst produces CO and CH_4_ from CO_2_ and water under UV–vis light irradiation, exhibiting a 42-fold photoactivity enhancement compared with g-C_3_N_4_ and nearly 100% selectivity towards CO_2_ reduction. Experimental and theoretical results reveal that the ionic liquid extracts electrons and facilitates CO_2_ reduction, whereas Co single atoms trap holes and catalyze water oxidation. More importantly, the maximum electron transfer efficiency for CO_2_ photoreduction, as measured with in-situ μs-transient absorption spectroscopy, is found to be 35.3%, owing to the combined effect of the ionic liquid and Co single atoms. This work offers a feasible strategy for efficiently converting CO_2_ to valuable chemicals.

## Abstract (web summary)

There is growing interest in designing photocatalysts for CO_2_ conversion. Here, the authors combine an ionic liquid with Co single atoms as dual-cocatalysts for g-C_3_N_4_, accelerating electron kinetics and improving CO_2_ reduction selectivity.

## Introduction

The worldwide consensus on carbon neutrality is driving the rapid development of technologies for CO_2_ conversions^1,2^. Recent research efforts have been devoted to photocatalytic CO_2_ conversion in pure water as a competitive route to produce valuable chemicals. However, it remains a huge challenge to construct efficient and stable photocatalytic systems^3^. Graphic carbon nitride (g-C_3_N_4_, CN), especially two-dimension nanosheets with controllable morphology, has emerged as a promising group of organic semiconductor photocatalysts, owing to its low cost, visible-light response, and robust nature. Notably, the negatively positioned conduction band (CB) imparts the photoelectrons of CN sufficient thermodynamic energy for the reduction of CO_2_. Nevertheless, its photocatalytic performance still suffers from fast charge carrier recombination as well as the lack of catalytic sites. Therefore, the photocatalyst design of CN-based photocatalysts is expected to prolong electron lifetime and improve CO_2_ reduction selectivity.

Loading suitable cocatalysts on CN is one of the most feasible strategies to simultaneously prolong the electron lifetime and introduce catalytic sites^4–6^. Ionic liquids (ILs) as a class of molten salts are composed of large, asymmetric organic cations and inorganic anions. The inherent features such as favorable dissolving/adsorbing ability, structural diversity, non-inflammability, wide electrochemical window, and high ion conductivity endow ILs with widespread applications^7,8^. Focusing on the typical imidazolium ILs, it is noted that the electronic structure of the imidazolium cation comprises a delocalized 3-center-4-electron conjunction across the N1-C2-N3 moiety, a double bond between C4 and C5 at the opposite side of the ring and a weak delocalization in the central region^9,10^. This would enable the cations to extract and stabilize electrons. Thus, it could be deduced that when using imidazolium IL as the surface modifier for a semiconductor, the imidazolium cation might extract electrons from the excited semiconductor. Moreover, imidazolium ILs have been comprehensively exploited to facilitate electrocatalytic CO_2_ reduction as appealing alternatives for conventional electrolytes/solvents^11^. Except for favorable CO_2_ adsorbing capability, research interest in imidazolium ILs mainly stems from their catalytic function for activating CO_2_^12–14^. Many relevant works highlight the role of imidazolium ILs as cocatalysts. For example, 1-ethyl-3-methylimidazolium tetrafluoroborate ([emim][BF_4_])^15^ was reported capable of reducing the initial barrier of CO_2_ conversion while suppressing the H_2_ evolution reaction, which improved the selectivity of electrochemical CO_2_ conversion^16^. Based upon the above analysis, loading a tiny amount of IL [emim][BF_4_] on CN as the cocatalyst could prolong the lifetime of electrons and enable selective CO_2_ reduction.

In another aspect, a simultaneous introduction of another cocatalyst for capturing the holes and catalyzing the water oxidation could further prolong the electron lifetime. As extensively explored cocatalysts in photocatalysis, transition metal oxides like Co oxide, etc., perform dual-functions of hole trapper and catalyst for water photooxidation^17,18^. Inspired by the hot concept of single-atom catalysis, tailoring nanoscale Co oxide to the O-coordinated Co single atoms would shorten the hole transfer distance hence accelerating the hole-trapping process^19^. Moreover, Co single atoms might promote the activation of water to facilitate hole-initiated water photooxidation^20^. Referring to the reported synthesis of borate-mediated O-coordinated Ni single atoms on CN^21^, it is feasible to construct O-coordinated Co single atoms on the borate-modified CN (Co-bCN) as cocatalyst.

To sum up, CN nanosheets co-loaded by IL [emim][BF_4_] and borate-anchored Co single atoms (IL/Co-bCN) are promising photocatalysts to realize high-efficiency selective CO_2_ reduction in water. Although in some works ILs have been utilized as surface modifiers^22^, combining IL with single-atom sites to construct a photocatalytic system is an interesting attempt. More importantly, it is critical to clarify the individual effects and potential synergistic effects of two cocatalysts/modifiers on electron kinetics for CO_2_ photoreduction. Transient absorption spectroscopy (TAS) is known as a powerful technique to elucidate the charge carrier kinetics for photocatalysts^23^. The multi-electron transfer involved in CO_2_ photoreduction occurs at the timescales of microseconds to seconds. Therefore, the μs-TAS under reaction conditions, namely in situ μs-TAS, is applicable for investigating the multi-electron reduction process, however, which remains unexplored.

Here, IL [emim][BF_4_] and borate-anchored Co single atoms were co-loaded on ultrathin CN nanosheets to obtain IL/Co-bCN nanocomposites, respectively forming spatially separated reduction domains and oxidation domains. The optimal nanocomposite applied in the CO_2_ photoreduction exhibited 9- and 42-fold CO_2_ conversion rates compared with those of bCN and CN, respectively, and provided nearly 100% selectivity towards CO_2_ reduction with CO and CH_4_ as products. IL was found capable of extracting electrons and enabling selective CO_2_ reduction, while Co single atoms were proved to trap holes and catalyze water oxidation. By in situ μs-TAS, the electron transfer efficiency (ETE) of IL/Co-bCN was quantified to be 35.3%. In addition, the linear correlation between electron transfer rate (ETR) and the loading amount of IL was discovered for the IL-catalyzed CO_2_ photoreduction. This work has provided an efficient approach for enhanced artificial photosynthesis and an investigation paradigm using in situ TAS.

## Results

### Synthesis of co-modified g-C_3_N_4_ nanosheets by IL and Co single atoms

**Fig. 1:**
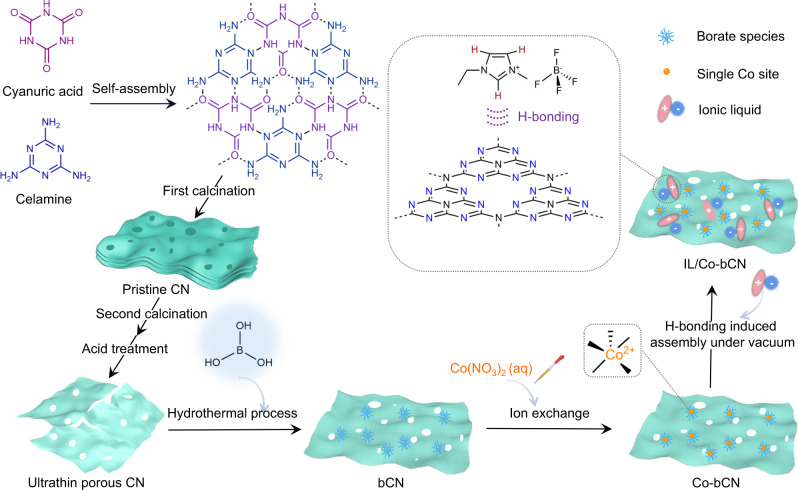
Illustration of the synthetic procedures of IL/Co-bCN photocatalysts. IL: ionic liquid 1-ethyl-3-methylimidazolium tetrafluoroborate ([emim][BF_4_]) is presented by pink oval cation and blue circle anion; bCN: boric acid modified ultrathin CN nanosheets, where borate species are presented by light blue urchin symbols; Co single atoms are presented by orange balls.

The IL/Co-bCN photocatalyst with dual-cocatalysts was synthesized as the procedures illustrated in Fig. 1. Using melamine and cyanuric acid as the raw material, pristine CN was fabricated by the H-bonding-induced self-assembling process followed by calcination. The second calcination with subsequent acid treatment for pristine CN resulted in ultrathin porous CN nanosheets (denoted as CN for short in the following discussion). With boric acid as the boron source, uniform borate species were modified on CN (bCN) driven by the dative B-N coordination through a low-temp hydrothermal process^21^. Next, the aqueous Co(NO_3_)_2_ solution was dropwise added into the aqueous bCN suspension under continuous stirring and ultrasound. As the previously reported synthetic method for Ni single atoms, the O-coordinated Co single atoms were constructed on bCN (Co-bCN) via the ion exchange between Co^2+^ with protons of supported borate species^21^.

On the other hand, since the hydrogen atoms bonding with the carbon atoms in [emim]^+^ are natural hydrogen donors^24^, they might form H-bonds with the *sp*^2^ nitrogen atoms of CN with large electronegativity. Accordingly, IL [emim][BF_4_] could be assembled on the exposed CN surface of Co-bCN to result in the IL/Co-bCN photocatalysts^25^, forming the separated reduction domains and oxidation domains.

### Photocatalytic CO_2_ conversion

**Fig. 2:**
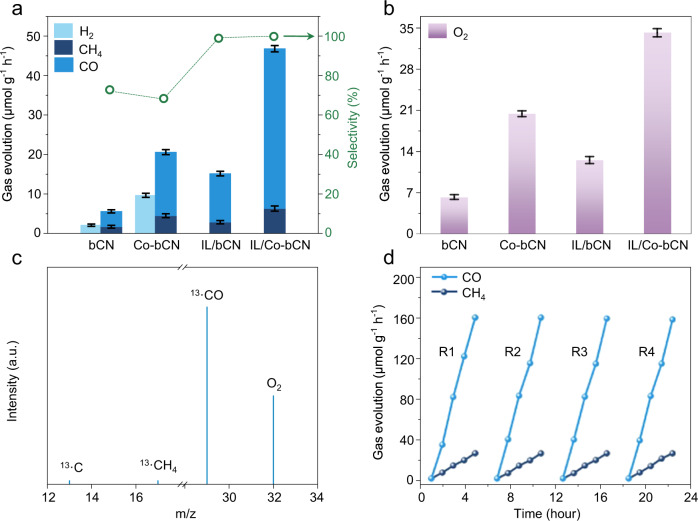
Photocatalytic CO_2_ conversion to valuable chemicals. **a** Photoreduction of CO_2_ and protons on bCN, IL/bCN, Co-bCN, and IL/Co-bCN under UV–vis light irradiation. Right Y-axis (green) indicates the product selectivity for CO_2_ photoreduction. **b** Oxygen evolution on bCN, IL/bCN, Co-bCN, and IL/Co-bCN during CO_2_ photoreduction. **c** Mass spectrum of the products from ^13^CO_2_ photoreduction by IL/Co-bCN. **d** Recycle test of IL/Co-bCN for the photocatalytic CO_2_ conversion. Data in panels a and b are presented as the mean ± standard deviation (s.d.) from three independent experiments.

The activities of as-synthesized samples were evaluated for the gas-phase CO_2_ photoreduction under irradiation by the 300 W Xenon lamp (Fig. 2a). By altering the boric acid amount, the best performance for bCN was obtained with CO, CH_4_, and H_2_ (3.9, 1.7 and 1.7 μmol g^−1^ h^−1^) as the reduction products (Supplementary Fig. 1a). After introducing Co single atoms, optimal Co-bCN was acquired with the largest O_2_ production rate (867.5 μmol g^−1^ h^−1^) and CO_2_ conversion rate (20.6 μmol g^−1^ h^−1^) for the photocatalytic water oxidation and CO_2_ reduction, respectively (Supplementary Fig. 1b and c). Moreover, compared with CoO_x_-CN obtained by directly loading Co-oxo species on CN, borate-mediated Co single atoms demonstrate superiority as cocatalysts. While although the introduction of Co single atoms could facilitate the CO_2_ photoconversion, H_2_ was still evolved (9.7 μmol g^−1^ h^−1^) as an undesired by-product. The loading of typical ILs like 1-ethyl-3-methyl imidazolium hexafluorophosphate ([emim][PF_6_]), 1-ethyl-3-methylimidazolium bis (trifluoromethyl) sulfonylimide ([emim][NTF_2_]), 1-butyl-3-methylimidazolium tetrafluoroborate ([bmim][BF_4_]), and [emim][BF_4_] on bCN resulted in a series of IL-modified bCN nanocomposites. The chemical structures of ILs are shown in Supplementary Fig. 2. All samples, especially the [emim][BF_4_] one, greatly enhanced the photoactivity for CO_2_ photoreduction (Supplementary Fig. 1d).

The best IL/bCN ([emim][BF_4_]-modified sample) produces CO and CH_4_ with production rates of 12.3 and 2.8 μmol g^−1^ h^−1^, respectively (Supplementary Fig. 1e). Next, adopting the most effective [emim][BF_4_] of different amounts to modify Co-bCN could obtain a series of co-modified samples. The best one, denoted as IL/Co-bCN for short in the following discussion, provides the CO and CH_4_ production rates of 40.5 and 6.3 μmol g^−1^ h^−1^, respectively, noteworthily without any H_2_ evolution (Fig. 2a and Supplementary Fig. 1f; corresponding gas chromatography raw data is included in Supplementary Fig. 3). This suggests the introduction of [emim][BF_4_] could improve CO_2_ reduction selectivity and inhibit H_2_ evolution, consistent with the discoveries in the IL-facilitated electrochemical CO_2_ reduction^11^. Remarkably, optimal IL/Co-bCN could provide 9- and 42-fold CO_2_ conversion rates of those for bCN and CN with nearly 100% selectivity towards CO_2_ reduction. Besides, all typical samples are found to produce approximately stoichiometric O_2_ without other oxidation products like H_2_O_2_, etc. (Fig. 2b), indicating a complete cycle of the photocatalytic reaction between CO_2_ and water.

To confirm that the reduction products be derived from CO_2_, the photoreduction of ^13^C-labeled CO_2_ was conducted over the IL/Co-bCN. The dominant peak of ^13^CO (*m/z* = 29) and a small peak of ^13^CH_4_ (*m/z* = 17) were observed in the selective ion detection chromatography spectrum (Fig. 2c, raw data in Supplementary Fig. 4), testifying the evolved CO and CH_4_ over IL/Co-bCN originate from the photoreduction of CO_2_. Moreover, four consecutive runs were then carried out over IL/Co-bCN and the photoactivity for each run shows a negligible decrease (Fig. 2d), reflecting the robust nature of IL/Co-bCN under reaction conditions. The superiority of IL/Co-bCN is then demonstrated by the comparison with the representative cocatalyst-involved CN-based photocatalysts for CO_2_ reduction (Supplementary Table 1). Obviously, IL/Co-bCN, exhibits the most competitive photoactivity especially in terms of apparent quantum yield (1.00%, 405 nm) and CO_2_ reduction selectivity, thanks to the co-loading of IL and Co single atoms.

### Structural characterizations

**Fig. 3:**
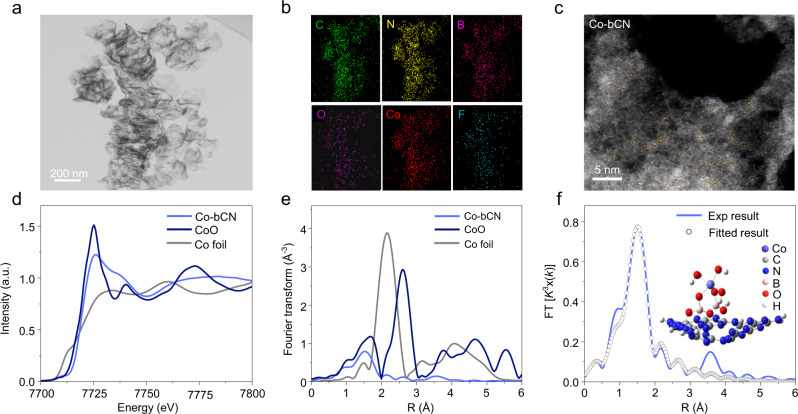
Characterization of IL/Co-bCN and Co-bCN. **a** TEM image of IL/Co-Bcn. **b** The corresponding EDX mapping images of elemental C, N, B, O, Co, and F of IL/Co-bCN. Scale bar: 200 nm. **c** The HAADF-STEM image of Co-bCN. Note: dots in orange circles indicate single Co atoms. Scale bar: 5 nm. **d** Normalized XANES spectra of Co-bCN, CoO, and Co foil, respectively, at the Co K-edge. **e** Fourier transformation of EXAFS spectra at the Co K-edge. **f** The corresponding EXAFS fitting curves of single-atom Co-bCN. Inset: simulated structure model of Co-bCN.

In the X-ray diffraction (XRD) patterns (Supplementary Fig. 5a), two characteristic diffraction peaks of CN at 13.1 and 27.3º are assigned to the in-planar repeated tri-s-triazine units (100) and the (002) interlayer stacking, respectively^26^. Compared with CN, bCN, and Co-bCN show no extra peaks due to either boron oxide or Co oxide, indicating the high dispersion of supported boron oxo- and Co species. For IL/Co-bCN, well-maintained peaks of CN indicate the introduction of IL does not change the crystalline structure of CN. The UV–vis diffuse reflectance spectra (DRS) show that neither IL nor Co species influence light absorption (Supplementary Fig. 5b). Besides, the specific surface area only decreases after loading IL due to the coverage (Supplementary Fig. 5c–f). Following this, the morphology of the samples was explored by transmission electron microscopy (TEM) and atomic force microscopy (AFM). As Supplementary Fig. 6a, CN takes on ultrathin silky nanosheets with random mesopores, in accordance with its specific surface area. For Co-bCN and CoO_x_-CN as a reference, no aggregates or particles could be observed in the TEM images (Supplementary Fig. 6b, c), indicating the high dispersion of both-type cobalt species. However, as shown by the photocatalytic performances, the borate-mediated Co single atoms are advantageous cocatalysts over the Co-oxo species in CoO_x_-CN. Compared with CN or Co-bCN (Supplementary Fig. 6d, e), the morphology and thickness of IL/Co-bCN (Fig. 3a and Supplementary Fig. 6f) remain nearly unchanged, once again proving the high dispersion of supported Co species and IL ion pairs on CN surface. The energy dispersive X-ray (EDX) mapping (Fig. 3b) images evidence the homogeneous state of C, N, B, O, Co and F elements, demonstrating the uniform distribution of borate species, Co species, and [BF_4_]^−^ anions on CN for IL/Co-bCN.

The atomic-resolution high-angle-annular-dark-field scanning transmission electron microscopy (HAADF-STEM) image of Co-bCN was collected to disclose the specific distribution status of Co species. The isolated dots marked by orange circles are attributed to Co single atoms, undoubtedly proving the atomic dispersion of Co species on bCN (Fig. 3c).

According to the position of the rising edge of the Co K-edge X-ray absorption near edge structure (XANES) spectra, the oxidation state of Co single atoms for Co-bCN was determined with CoO and Co foil as contrast (Fig. 3d). The spectral line shape and the absorption edge position resemble those of CoO, indicating Co single atoms are in the valance of around +2. The Fourier transform of the extended X-ray absorption fine structure (EXAFS) spectra in Fig. 3e demonstrates that no first-shell Co-Co contribution exists, evidencing the single-atom state of Co species instead of metallic Co nanoparticles or clusters. The EXAFS data fitting results indicate the main peak is at about 2.0 Å, which value is close to the Co-O bond length of CoO (Fig. 3f and Supplementary Table 2)^27,28^. In addition, the coordination number of the Co single atom is determined to be five. Accordingly, the theoretical model of single Co sites was simulated and shown in Fig. 3f inset (bond lengths are shown in Supplementary Fig. 7a)^29^. To be specific, the O atoms of borate species along with water and hydroxyl groups coordinate with the Co atom, resulting in a five-O-coordinated Co single-atom site, which is denoted as a single Co (II)-O_5_ site. Interestingly, the brightness of individual dots on IL/Co-bCN in the HADDF-STEM image (Supplementary Fig. 7b) maintains, implying the introduction of IL does not influence the distribution of Co single atoms. This might be on account of the as-proposed separated domains for IL and Co single atoms on the CN surface. Besides, the chemical states of typical samples were also characterized by the X-ray photoelectron spectra (XPS) (Supplementary Fig. 8 and Supplementary Table 3). In the Co 2*p* spectra, similar to the Co 2*p* peaks of CoO_x_-CN, the Co 2*p*_1/2_ (795.8 eV) and Co 2*p*_3/2_ (780.3 eV) peaks with their satellites for Co-bCN are attributed to the oxidized cobalt in +2 valence, which is consistent with the XANES results^30^. By contrast, the binding energy (BE) of the Co 2*p* peaks of IL/Co-bCN shows a negligible shift, indicating IL do not affect the chemical state of Co single atoms. Moreover, the atomic concentration of Co for Co-bCN and IL/Co-bCN are almost identical, which further evidences the loading of IL does not affect Co single-atom sites (Supplementary Table 3). The F 1*s* peaks of IL/bCN and IL/Co-bCN locate at the same BE, attributed to the [BF_4_]^−^ anion, suggesting IL ions are successfully introduced on CN and no strong chemical interaction exists between the [BF_4_]^−^ anions and Co single atoms.

To validate the inferred H-bonding interaction between IL and CN in IL/Co-bCN, a simplified IL/CN model was set up by the molecular dynamics (MD) simulation. The simulation results reveal that the IL/CN system is stable (Supplementary Fig. 9a–d). The H-atoms bonding to C2, C4, and C5 (Supplementary Fig. 9e and f) in the [emim]^+^ ring could form H-bonds with the *sp*^2^ N atoms of CN. Despite the H-bonding interaction between [emim]^+^ and borate species might exist as the simulated IL/bCN model (Supplementary Fig. 10), the interaction between IL and CN in IL/Co-bCN is predominantly via the H-bonding between the [emim]^+^ and CN due to the advantageous dispersion rate of *sp*^2^ N atoms over the borate species.

### Roles of IL and Co single atoms

The effects of IL and Co single atoms on the charge carrier properties were investigated by the photochemical and photophysical techniques. In the electron paramagnetic resonance (EPR) spectra under illumination, the signal intensities from 5, 5-dimethyl-l-pyrroline N-oxide (DMPO)-·OH adducts, where DMPO is used as the spin-trapping agent, are found to increase as the same order with the photocatalytic activities (bCN < IL/bCN < Co-bCN < IL/Co-bCN) (Supplementary Fig. 11a). Generally, the ·OH radicals are produced by photooxidation of water, hence which amount could reflect the efficiency of charge separation. Therefore, the EPR spectra for detecting DMPO-·OH adducts indicate that either IL or Co single atoms could improve the charge separation and the combination of two modifiers for IL/Co-bCN leads to the maximum improvement. Moreover, steady-state photoluminescence (PL) spectra and electrochemical impedance spectroscopy (EIS) agree well with the above conclusion (Supplementary Fig. 11b and c). To further distinguish the individual modulating features of IL and Co single atoms on the charge carriers of CN, the atmosphere-control surface photovoltage spectroscopy (AC-SPS) was performed on bCN, IL/bCN, and Co-bCN^31^. As Supplementary Fig. 12a, a negligible SPS signal of bCN was detected in the atmosphere of N_2_ due to the intrinsic poor charge separation. The signal intensities of IL/bCN in different atmospheres decrease as the order of O_2_ > air > N_2_, where O_2_ shows a positive effect on the charge separation. Since O_2_ is a well-known electron scavenger^32^, it can be inferred IL attract the photoelectrons to improve the charge separation (Supplementary Figure 12b)^7^. For Co-bCN, the signal intensities in different atmospheres decrease as the order of N_2 _> air > O_2_, as opposed to that for IL/bCN, where O_2_ is found to impose a negative effect. This suggests that Co single atoms trap holes instead of electrons to facilitate the charge separation (Supplementary Fig. 12c)^33^.

**Fig. 4:**
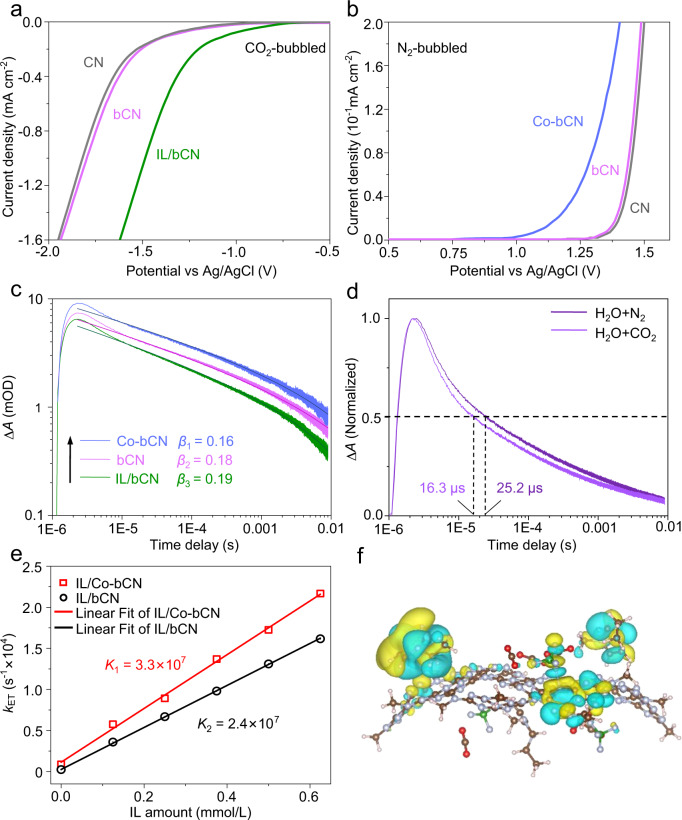
Roles of IL and Co single atoms. **a** Electrochemical reduction curves of CN, bCN, and IL/bCN in the CO_2_-bubbled system. **b** Electrochemical oxidation curves of CN, bCN, and Co-bCN in the N_2_-bubbled system. **c** Fitted parameter for an assumed power-law decay (*I*_TAS_∝(*t*-*t*_0_)^−β^) of bCN, Co-bCN, and IL/bCN in the gaseous mixture of N_2_/H_2_O vapor, excited by pulsed 355 nm and monitored at 900 nm. **d** In situ μs-TAS spectra over IL/Co-bCN in the gaseous mixture of N_2_/H_2_O vapor or CO_2_/H_2_O vapor, excited by pulsed 355 nm and monitored at 900 nm. **e** Electron transfer rates (ETRs) versus IL amount on IL/bCN (black circle) and IL/Co-bCN (red square). The rate constant is obtained from the slope of the linear fit, whereas the background rate is taken from the intercept. **f** Differential charge density diagram of the CO_2_ + IL/CN system with isosurface value of 0.003 e/Å^3^.

As another key factor, the catalytic functions of IL and Co single atoms, significantly determining catalytic efficiencies and selectivity, were investigated by collecting the electrochemical reduction/oxidation curves. In the electrochemical reduction system bubbled with CO_2_, IL/bCN shows the smallest onset potential for CO_2_ reduction, evidencing IL exerts the catalytic ability to activate CO_2_ molecules (Fig. 4a). This agrees with the reported catalytic effect of IL in electrocatalytic CO_2_ reduction^34^. While in the electrochemical oxidation system bubbled with N_2_, Co-bCN displays the smallest oxidation onset potential, confirming that Co single atoms could catalyze water oxidation (Fig. 4b). Accordingly, the enhanced photoactivity of IL/Co-bCN originates from the combined effect of IL for extracting electrons as well as catalyzing selective CO_2_ reduction and Co single atoms for capturing holes as well as catalyzing water oxidation.

To acquire a deep insight into the photocatalytic reaction mechanism over IL/Co-bCN, μs-TAS was employed to study the charge dynamics (Supplementary Fig. 13). The positive TAS signals of CN spanning the entire visible and near-IR regions are attributed to photoexcited electrons, while the hole contribution to the TAS signal is negligible^35,36^. Therefore, it is feasible to directly monitor the electron kinetics of CN but to indirectly observe hole transfer also by detecting the electron signal. The wavelength-dependent TAS signal of CN was measured with the addition of triethanolamine (TEOA) as a well-known efficient hole scavenger in order to verify the electron fingerprint regions^35,36^. Considering the TAS signal intensity increases as the wavelength, 900 nm as the fingerprint of the electrons was selected to be the detecting wavelength (Supplementary Fig. 14). As Fig. 4c, the μs-TAS curves for electron decay kinetics of bCN, Co-bCN, and IL/bCN at 900 nm are well fitted by a power law function of *I*_TAS_(*t*)∝(*t*-*t*_0_)^−*β*^, where *β* (0 < *β* *<* 1) represents the fitting stretching exponent, as evidenced by the linear decay on a log–log plot with a trapping-detrapping dynamics^23,35–38^. The fitting yields a *β* value of bCN, which is between those of IL/bCN and Co-bCN. Moreover, the half-lifetime denoted as *t*_50_ is defined as the time when 50% of the initial excited state population decayed by half, representing the average kinetic decay constant of the TAS curves. Therefore, it is reasonable to employ *t*_50_ to evaluate the electron kinetic decay process^37,38^. The intrinsic decay of bCN yields a representative electron *t*_50_ of 29.1 μs. Compared to bCN, the TAS amplitude of IL/bCN at the initial time decreases from 0.007 to 0.006 with a shorter electron lifetime (*t*_50_ = 22.5 μs), suggesting that electron transfer from bCN to IL. In contrast, Co-bCN shows higher TAS amplitude than that of bCN (increasing from 0.007 to 0.009) with a longer electron lifetime (*t*_50_ = 37.6 μs), inferring holes are trapped by Co single atoms. To evaluate the hole-trapping ability of Co single atom, the electron decay kinetics of Co-bCN was compared with that of bCN with methanol (MeOH) added as an efficient hole scavenger (the moles of MeOH are identical with those for cobalt in Co-bCN). It is observed Co-bCN (*t*_50_ = 37.6 μs) exhibits a longer *t*_50_ than bCN with MeOH added (*t*_50_ = 31.9 μs) (Supplementary Fig. 15), which suggests that Co single atoms possess a stronger ability to extract holes than MeOH. Overall, the modulating features of IL and Co single atoms were verified to be effective electron extractors and hole trappers, respectively.

Following the charge dynamics observation, the subsequent issue lies in understanding the kinetics mechanism of CO_2_ photoreduction, which is crucial to designing photocatalytic materials. By far, the kinetic mechanisms of CO_2_ photoreduction remain unclear, due to the inadequate fundamental understanding of charge carrier kinetics in a real reaction system. In situ μs-TAS is a powerful technique to elucidate the charge carrier kinetics of the photocatalysts by monitoring the reaction process^39^. Time-dependent observation of CO_2_ reduction could reveal insight into the depopulation mechanisms of the charge state of samples. The in situ TAS kinetics for specific samples was collected by imputing gaseous mixture of CO_2_/H_2_O vapor into the sample cell to mimic the CO_2_ reduction conditions, respectively. Besides, the measurements by imputing gaseous mixture of N_2_/H_2_O vapor were performed as a contrast. The TAS signal at 900 nm measures the population of the electron state that decays following the power law to the ground state through radiative and non-radiative processes with the rate of 1*/t*_50_ (N_2_) = *k*_R + _*k*_NR_ and 1*/t*_50_ (CO_2_) = *k*_R + _*k*_NR + _*k*_ET_ in the presence of N_2_/H_2_O vapor and CO_2_/H_2_O vapor, respectively, where *k*_R_ and *k*_NR_ were introduced as the intrinsic radiative rate and nonradiative rate on bCN, respectively, and the *k*_ET_ for the ETR, which indicates the electron transfer rate from the photocatalyst to CO_2_. The electron transfer efficiency (ETE) during the CO_2_ reduction is expressed by the following equation:^40–42^

1 $$\,{{{{{\rm{\eta }}}}}}=1-\frac{{{{{{t}}}}}_{50}({{{{{{\rm{CO}}}}}}}_{2})}{{{{{{t}}}}}_{50}({{{{{{\rm{N}}}}}}}_{2})}$$

Based on Eq. (1), it can be inferred that boosting the ETE for CO_2_ reduction, which describes the effectiveness of the electron transfer processes, can be achieved by facilitating the electron capture by CO_2_ on the basis of the prolonged electron lifetime of bCN by hole trapping. The ETE for CO_2_ reduction was calculated based on Eq. 1 and presented in Supplementary Fig. 16. The ETE of bCN is as low as ~0.7%, indicating that CO_2_ could hardly capture electrons from bCN. The TAS decay of IL/bCN distinctly becomes fast with a *t*_50_ of ∼16.5 μs in the presence of CO_2_, indicating that IL open the channel for CO_2_ to capture electrons and serve as good catalytic activation sites for CO_2_ reduction. While after the Co single-atom modification, although the electron lifetime was prolonged, the ETE of ~3.2% is still limited. By contrast, the maximum ETE reaches ∼35.3% for IL/Co-bCN as calculated according to the TAS kinetics in Fig. 4d, which is around 93-fold of that for CN (0.3%). Therefore, the photoactivity enhancement of IL/Co-bCN is attributed to the high ETE contributed by IL based on prolonged electron lifetime due to the hole-trapping function of Co single atoms.

To elucidate IL-catalyzed CO_2_ reduction kinetics, the quantitative effect of IL was examined on the ETR, which was calculated based on the formula of *k*_ET_ = 1/*t*_50_(CO_2_) − 1/*t*_50_(CO_2_)^41,42^. Based on the above TAS results, IL has been verified to function as a favorable electron acceptor and also a catalyst for CO_2_ reduction, however, which has seldom been investigated in photocatalysis. Thus, the quantitative relationship between the loading amount of IL and the corresponding ETR was further explored. The ETR values on the *x*IL/bCN and *x*IL/Co-bCN, where *x* indicates the loading amount of IL, are calculated based on the analysis of their TAS decay curves (Supplementary Figs. 17 and 18), and summarized in Supplementary Tables 4 and 5, respectively. For IL/bCN, the ETR increases with the IL amount, indicating that the IL modification greatly accelerates electron transfer kinetics (Fig. 4e). Moreover, the ETR as a function of the IL amount can be well fitted by the first-order kinetics of *k*_ET_ = *Kc*^43,44^, where *c* and *K* represent the IL amount and the rate constant, respectively, implying the photocatalytic CO_2_ reduction with IL as the catalytic sites might obey the first-order principle. The fitting yields a large rate constant of 2.4 × 10^7^ M^−1^ s^−1^ on IL/bCN photocatalyst. Besides, the kinetic analysis also informs us of a slow background reaction rate *k*_0_ of 238 s^−1^, due to the weak CO_2_ activation in the absence of IL. By the co-modification of IL and Co single atoms, the reaction rate constant increases up to 3.3 × 10^7^ M^−1^ s^−1^, owing to prolonged electron lifetime and the promoted CO_2_ activation by the synergistic effects of two cocatalysts.

Moreover, the theoretical analysis by combining the density functional theory (DFT) calculation and MD simulation was performed to further validate the modulating features of two cocatalysts. For the simulated Co-bCN model (Supplementary Fig. 19a and b), the differential charge density diagram and hole distribution of excited Co-bCN (Supplementary Fig. 19c and d) indicate the region around Co single atoms is in positive charge after excitation, proving Co single atoms can trap holes. On the other hand, the electrons of the IL/CN system predominantly distribute at the [emim]^+^ ring and the IL/CN interface, indicating ILs might extract the photoelectrons (Supplementary Fig. 9c and d). Furthermore, when CO_2_ exists, the direct adsorption of CO_2_ on CN is found unstable (Supplementary Fig. 20). While for the equilibrium geometry of the CO_2 _+ IL/CN system, the CO_2_ molecule tends to locate near the bonding region between IL and CN. By comparing the adsorption distance and interaction energy, the CO_2_ adsorption for the CO_2 _+ IL/CN system is more stable than that for the CO_2_ + CN system (Supplementary Fig. 21). The electron density of the CO_2 _+ IL/CN system shows that the electron populations are larger on the side of [emim]^+^, again manifesting IL could extract electrons (Fig. 4f).

**Fig. 5:**
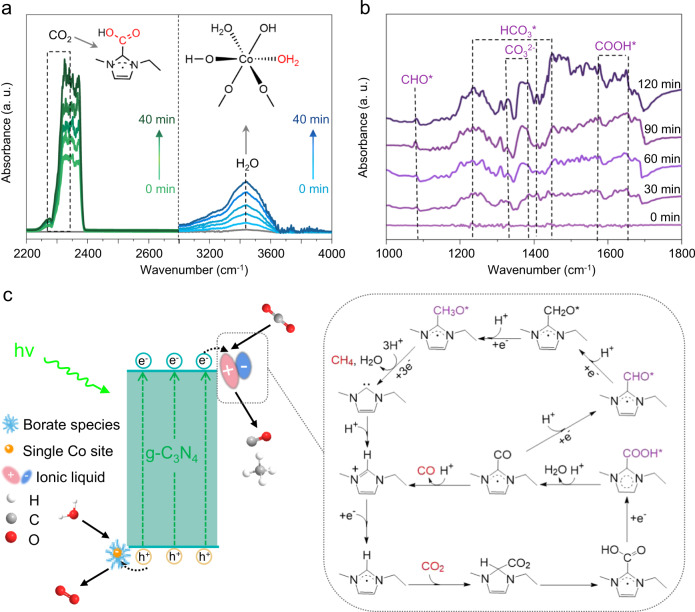
Mechanism for CO_2_ photoreduction on IL/Co-bCN. **a** FTIR spectra for adsorption of gaseous mixture of CO_2_/H_2_O vapor on IL/bCN (left panel) and Co-bCN (right panel) in dark, respectively. **b** In situ FTIR spectra for the CO_2_ photoreduction process on IL/Co-bCN. **c** Illustration of the proposed photocatalytic mechanism of CO_2_ photoconversion over IL/Co-bCN under UV–vis light irradiation.

To uncover the whole photocatalytic reaction mechanism, especially the catalytic reduction process, the adsorption of reactants was examined on typical samples by FTIR. The saturated adsorption spectra of the gaseous CO_2_/H_2_O vapor mixture for bCN, IL/bCN, and Co-bCN were separately collected under dark conditions (Supplementary Fig. 22a–c, Fig. 5a). Generally, the bands at 2350 and 3400 cm^−1^ demonstrate the adsorption of CO_2_ and water, respectively. The adsorption capacity for CO_2_ on IL/bCN (Fig. 5a, the left panel) is greatly enhanced compared with bCN or Co-bCN. This is because CO_2_ is prone to bind with imidazolium cations to form adducts, which has been comprehensively reported^12–14^. Compared with bCN and IL/bCN, Co-bCN shows an obviously enlarged peak area (Fig. 5a, the right panel), demonstrating promoted adsorption capacity of water over Co single atoms. Therefore, IL/Co-bCN with both cocatalysts shows the simultaneously promoted adsorption with CO_2_ and water, providing the foundation for efficient photocatalytic conversion.

Furthermore, to detect the intermediates involved in the photocatalytic CO_2_ reduction catalyzed by IL/Co-bCN, in situ FTIR measurements were performed on typical samples under reaction conditions. The infrared peak intensities of bCN show minor changes as the extension of the irradiation time, reflecting its weak photoactivity for CO_2_ reduction (Supplementary Fig. 22d). For IL/bCN and Co-bCN, the most remarkable difference between the two individual modified samples is that the peak intensity of COOH* regarded as the key intermediate for CO_2_ reduction becomes larger when IL is involved (Supplementary Fig. 22e and f).

For IL/Co-bCN, the peak appearing at 1080 cm^−1^ is ascribed to CHO*, which is the intermediate correlated with the formation of CH_4_^45,46^. The intermediates including CO_3_^2−^ (1326 and 1382 cm^−1^), HCO_3_* (1243, 1408, and 1458 cm^−1^) and COOH* (1554 and 1638 cm^−1^) are also detected (Fig. 5b). For the identical duration, the peak areas of IL/Co-bCN assigned to HCO_3_*, CO_3_^2−^ and COOH* are obviously the largest among all tested samples, indicating the advantage of bi-functionalization on the photocatalytic CO_2_ reduction. In addition, the in situ FTIR spectra using ^13^CO_2_ instead of ^12^CO_2_ further verified the production of key intermediates as mentioned above, supporting the speculative photocatalytic CO_2_ reduction process mechanism on IL/Co-bCN (Supplementary Fig. 23).

## Discussion

On the basis of the above results and analysis, a possible mechanism for CO_2_ photoreduction over IL/Co-bCN is proposed in Fig. 5c. Under UV–vis light irradiation, Co single atoms would trap holes and catalyze water oxidation, which prolongs the electron lifetime. While the [emim]^+^ cations on CN can extract photoelectrons to further prolong their lifetime and catalyze CO_2_ reduction. For the specific CO_2_ reduction process catalyzed by imidazolium ILs, it is proposed that carboxylation at the C2 position of imidazolium cations would occur, which is triggered by the reaction with CO_2_. The formation of the corresponding CO_2_-imidazolium adduct has been verified by ^13^C-nuclear magnetic resonance (NMR) in some works^47–53^. In addition, C4 and C5 atoms are also possible binding positions for CO_2_^54^. Combining these discoveries and the detected intermediates by in situ FTIR, the CO_2_ reduction pathway with [emim]^+^ as cocatalyst was illustrated, where the C2 atom of [emim]^+^ is showcased as the binding position. In particular, CO_2_ would bind with the C2 atom of N-heterocyclic carbene intermediate derived from reduced [emim]^+^ by one electron, resulting in the CO_2_-[emim] adduct. A subsequent reduction of the CO_2_-[emim] adduct by one electron leads to the formation of the COOH*-[emim] adduct, which is then attacked by one proton to generate the CO-[emim] adduct and one water molecule. Eventually, the attack by one proton enables the production of CO meanwhile [emim] turns back to [emim]^+^. As supplementary, the minor portion of CO-[emim] adduct is inferred to be attacked by continuous multiple electrons and protons to produce CH_4_^55^. In addition, IL might generate a positively charged interfacial layer to repel protons hence inhibiting the H_2_ evolution^56^.

To summarize, co-loading of IL [emim][BF_4_] and borate anchored Co (II)-O_5_ single sites on CN resulted in IL/Co-bCN photocatalysts, forming spatially separated reduction domains and oxidation domains. The optimized photocatalyst realized 42-fold CO_2_ conversion rate of that for CN and nearly 100% selectivity towards CO_2_ reduction. IL was evidenced to attract electrons and facilitate CO_2_ reduction, whereas Co single atoms could extract holes and catalyze water oxidation, supported by experimental results and theoretical simulation. Employing in situ μs-TAS, the ETE on IL/Co-bCN for photocatalytic CO_2_ reduction was quantified to be 35.3%, which is 93-fold of that for CN. The effectively accelerated electron kinetics is attributed to the combined effect of IL and Co single atoms. Moreover, the linear correlation between IL loading amount and ETR is discovered for the IL-catalyzed CO_2_ photoreduction. Thus, this work provides a comprehensive insight into electron kinetics for photocatalysis.

## Methods

### Material preparation

All chemicals and reagents were of analytical grade and used without further purification, which can be found in Supplementary Information (Material preparation). Deionized water used in all the experiments has a resistivity of 18.1 MΩ cm.

### Synthesis of bulk CN

Melamine (10 g) and cyanuric acid (4 g) were dispersed in deionized water (500 mL) and mixed under continuous stirring at 80 °C for 3 h. The mixture was cooled to room temperature, centrifuged at 4000 rpm for 5 min, and then dried at 80 °C for 12 h. The as-obtained powder was calcinated in a tube furnace from room temperature to 520 °C at a rate of 1 °C min^−1^ for 4 h under the N_2_ atmosphere to obtain bulk g-C_3_N_4_ (bulk CN).

### Synthesis of ultrathin CN

Bulk CN underwent two-time calcination in the semi-closed porcelain boat at 500 °C for 2 h in the air atmosphere. After natural cooling to room temperature, the as-obtained fluffy product was treated with HNO_3_ solution (5 mol L^−1^) and then dried for 12 h in the vacuum at 80 °C to obtain ultrathin CN.

### Synthesis of boric acid modified CN (bCN)

Ultrathin CN (0.5 g) was dispersed in deionized water (30 mL) under stirring and ultrasound for 1 h to result in an aqueous CN suspension. A desired volume of aqueous boric acid solution (0.05 mol L^−1^) was added into the CN suspension followed by stirring and ultrasound for 3 h. The as-obtained mixture was further transferred into a stainless-steel vessel and kept at 120 °C for 2 h. After cooling to room temperature naturally, the solid powder was centrifuged (8000 rpm), washed with absolute ethanol, and then dried in the vacuum at 60 °C. The samples were represented by *x*bCN, in which *x*% indicates the mass ratio (9, 12, and 15%) of boric acid relative to CN.

### Synthesis of Co-single-atom modified bCN (Co-bCN)

As-obtained bCN (0.2 g) was dispersed in deionized H_2_O (100 mL) under stirring and ultrasound for 0.5 h to result in aqueous bCN suspension. A certain volume of aqueous Co(NO_3_)_2_ solution was dropwise added into the bCN suspension. The as-obtained liquid mixture was stirred in the water bath at 80 °C for 3 h. Finally, the solid powder was centrifuged (8000 rpm), washed with absolute ethanol thoroughly, and dried in the vacuum at 60 °C. The samples were represented by *y*Co-bCN, where *y* *×* 10^−5^ indicates the concentration (1.0 × 10^−5^, 1.5 × 10^−5^, and 2.1 × 10^−5^ mol L^−1^) of Co(NO_3_).

### Synthesis of IL-modified bCN (IL/bCN)

An even mixture of [emim][BF_4_]and MeOH with an IL concentration of 5 mmol L^−1^ was prepared. As-obtained bCN (0.05 g) was introduced in the IL/ MeOH solution of the desired volume to result in uniform suspension under ultrasound conditions. Afterward, the suspension was dried in a vacuum oven at 80 °C for 12 h. As-synthesized samples were denoted as *m*IL/bCN, where *m* indicates the concentration (0.5, 0.625, and 0.75 mmol L^−1^) of IL. For the modification of other ILs ([emim][PF_6_]), [bmim][BF_4_], and [emim][NTF_2_]), identical synthetic procedures were performed as above.

### Synthesis of IL-modified Co-bCN (IL/Co-bCN)

The synthetic procedure is the same as that for IL/bCN by replacing bCN with optimal Co-bCN. As-synthesized samples were denoted as *n*IL/Co-bCN, where *n* indicates the concentration (0.5, 0.625, and 0.75 mmol L^−1^) of IL.

### Characterisations

The X-ray powder diffraction (XRD) patterns of the samples were measured with a Bruker D8 Advance diffractometer, using Cu Ka radiation. The UV–vis absorption spectra of the samples were measured with a Model Shimadzu UV2550 spectrophotometer, using BaSO_4_ as a reference. The Fourier transform infrared (FTIR) spectra of the samples were collected with a Bruker Equinox 55 Spectrometer, using KBr as diluents. The FTIR spectra in the figures have been normalized to the background before and after the main features. The X-ray photoelectron spectroscopy (XPS) technique was used to detect the surface composition and elemental chemical state of the samples, by using a Model VG ESCALAB apparatus with an Mg K X-ray source, and the binding energies were calibrated with respect to the signal for adventitious carbon (binding energy = 284.6 eV). The morphology of the samples was analyzed by transmission electron microscopy (TEM) on the FEI Tecnai G2 S-Tw in an instrument with an acceleration voltage of 200 kV. The thickness of the samples was analyzed by Atomic force microscopy (AFM) on a multimode nanoscope VIII instrument (Bruker) with mica as the base. The high-angle annular dark field scanning transmission electron microscopy (HAADF-STEM) images were collected on the FEI Titan 60–300 electron microscope equipped with a spherical aberration corrector. X-ray absorption spectroscopy (XAS) measurements were conducted at BL12B1 of Taiwan Beam Line in the SPring-8. The electron-storage ring was operated at 8 GeV with a current of 100 mA. A Si (111) double-crystal monochromator was employed for the energy selection with the resolution d*E*/*E* better than 2 × 10^−4^ at elemental edges. All XAS spectra were recorded at room temperature in fluorescence (for sample) and transmission (for reference) mode.

### Photocatalysis measurements

For the photocatalytic CO_2_ reduction test, the powder sample (0.05 g) was dispersed on filter paper contained in a cylindrical quartz reactor. Pure water (3 mL) was added below the holder, providing humidity while avoiding direct contact with the samples. The reactor system was then filled with the gaseous mixture of CO_2_/H_2_O by bubbling pure CO_2_ (15 bar) through deionized water following the complete evacuation and equilibrated for 20 min. When the adsorption reached equilibrium, the reactor system was irradiated with the 300 W Xenon lamp as the light source and the light output power was measured by a MEIKONG TN99D calibrated photodetector. Meanwhile, the circulating water was applied to control the temperature of the photocatalytic reduction at room temperature. The environment temperature (25 ^o^C) and relative humidity (50%) were controlled to be constant by the air conditioner. The gaseous product mixture (0.25 mL) was withdrawn from the headspace of the reaction cell at given time intervals for the quantitative analysis of carbon-containing reduction products by the gas chromatograph (GC-7920, CEAULIGHT) with flame ionization detector (FID). Similarly, the gaseous mixture (0.25 mL) was also withdrawn for the quantitative analysis of produced H_2_ and O_2_ during the CO_2_ photoconversion by the gas chromatograph (GC-2002, KE CHUANG) equipped with the thermal conductivity detector (TCD). The isotopic experiment also followed similar procedures, but CO_2_ was replaced by ^13^CO_2_ (Yuejia Gas, Guangdong, China). The products were detected by the gas chromatography-mass spectrometer (GC-7890B/MS5977A, Agilent, America). For the photocatalytic water oxidation test for *y*Co-bCN, 50 mg of photocatalyst powder was well dispersed in an aqueous solution (100 mL) containing AgNO_3_ (0.01 mol L^−1)^ as an electron scavenger and La_2_O_3_ (0.2 g) as a pH buffer agent. The gaseous product (0.25 mL) was withdrawn for the quantitative analysis of O_2_ also by the gas chromatograph (GC-2002, KE CHUANG) equipped with the thermal conductivity detector (TCD).

## Supplementary information

Supplementary information

## Data Availability

The authors declare that the data that supports the findings of this manuscript can be found in the Supplementary Information and are available free of charge or available from the corresponding author upon request. The source data underlying Figs. 2a–d, 3d–f, 4a–e and 5a, b on the experimental as well as the source data underlying Fig. 4f on the theoretical simulation of CO_2_ + IL/CN system are provided as a Source Data file. Source data are provided with this paper.

## Source data

Source Data
