# Supplementary material for: Improving CO2 photoconversion with ionic liquid and Co single atoms
Source: Nat Commun. 2023 Mar 16;14:1457. doi: 10.1038/s41467-023-36980-5 (PMC10020152; doi:10.1038/s41467-023-36980-5)
Supplement: Supplementary file 1 — Supplementary information [file 41467_2023_36980_MOESM1_ESM.pdf]

Supplementary Information

Improving CO_2 photoconversion with ionic liquid and Co single atoms

Liu et al.

Improving CO_2 photoconversion with ionic liquid and Co single atoms

Yang Liu[1], Jianhui Sun[1,2], Houhou Huang[3], Linlu Bai[1, *], Xiaomeng Zhao[1], Binhong Qu[1], Lunqiao Xiong[4], Fuquang Bai[3], Junwang Tang[4, *], Liqiang Jing[1, *]

Author affiliations

1. Department Key Laboratory of Functional Inorganic Materials Chemistry (Ministry of Education), School of Chemistry and Materials Science, International Joint Research Center and Lab for Catalytic Technology, Heilongjiang University, Harbin, Heilongjiang 150080 (P. R. China).
2. Department Key Laboratory of Functional Inorganic Materials Chemistry (Ministry of Education), International Joint Research Center for Catalytic Technology, School of Physics, Heilongjiang University, Harbin, 150080 (P. R. China).
3. International Joint Research Laboratory of Nano-Micro Architecture Chemistry, Institute of Theoretical Chemistry and College of Chemistry, Jilin University Changchun 130021 (P. R. China).
4. Department of Chemical Engineering, University College London Torrington Place, London WC1E 7JE (UK).

To whom correspondence should be addressed. E-mail: llbai@hlju.edu.cn (L. B.); junwang.tang@ucl.ac.uk (J. T.); jinglq@hlju.edu.cn (L. J.).

Table of contents

Supplementary Computation Section 3
Supplementary Methods 3
Sample characterization 4
Supplementary Figure 1 7
Supplementary Figure 2 8
Supplementary Figure 3 9
Supplementary Figure 4 10
Supplementary Figure 5 11
Supplementary Figure 6 12
Supplementary Figure 7 13
Supplementary Figure 8 14
Supplementary Figure 9 15
Supplementary Figure 10 16
Supplementary Figure 11 17
Supplementary Figure 12 18
Supplementary Figure 13 19
Supplementary Figure 14 20
Supplementary Figure 15 21
Supplementary Figure 16 22
Supplementary Figure 17 23
Supplementary Figure 18 24
Supplementary Figure 19 25
Supplementary Figure 20 26
Supplementary Figure 21 27
Supplementary Figure 22 28
Supplementary Figure 23 29
Supplementary Table 1 30
Supplementary Table 2 33
Supplementary Table 3 34
Supplementary Table 4 35
Supplementary Table 5 36
Supplementary Reference 37

Supplementary Computation Section

In this work, the conformation of all systems was optimized via density functional theory (DFT) calculation[1]. In order to get more reasonable g-C_3N_4 nanosheets (CN) conformation, nine CN units form five holes to adsorb CO_2 and ionic liquid (IL) [emim][BF_4]. The solvation structures of CN−solvent−anion complexes, electrolyte, and CO_2 adsorption were simulated through the molecular dynamics (MD) simulations. The parameters of porous CN were from All-atom automatic OPLS-AA topology generator, lacked parameters were added by empirical data. Parameters of $[emim]^+$ and BF_4^- were obtained from All-atom automatic OPLS-AA topology generator as well, Part of lacked parameters were from previous job[2]. Parameters of CO_2 were come from OPLS-AA force field[3]. Then, three different molecule dynamics simulation systems were built by PACKMOL software package[4]. In the first molecule dynamics simulation system, six CO_2 molecules with one porous CN material were set in a 28 nm × 18 nm × 32 nm box. In the second, one porous CN material with three $[emim]^+$ and $[BF_4]^-$ were added in a 26 nm × 17 nm × 30 nm box. In the third, one porous CN material, six CO_2 molecules, three $[emim]^+$ and three $[BF_4]^-$ ions. Box size of each system was built as small as possible to reduce computational cost. Molecule dynamics simulations were performed with the GROMACS 5.1.4 software package[5]. Before MD simulations were performed, each of systems was executed with 100,000 steepest descent steps to prevent steric clashes or inappropriate geometries. The cut-off distance of short Coulomb and Van der Waals was set to 0.8 nm, respectively. To reduce the movement of CN material, each of C and N atom was fixed in the system position with force constants of 1000 kJ mol^{-1} nm^{-2}. The V-rescale method was used as a temperature coupling type to heat to 300 K[6]. MD simulations were performed under NPT ensemble constant temperature with 20 ns. After the system was equilibrated, some snapshots of trajectories were further analyzed. For the snapshot extracted by random average, Grimme's DFT-D2 dispersion correction method was employed to describe the vdW interactions[7]. The characteristics of hydrogen bond, differential charge density, and electrostatic potential (isosurface value 0.003 e/$Å^3$) obtained from the calculation of equilibrium structure are analyzed and presented in the text.

Supplementary Methods

Material preparation. Melamine (Aladdin, AR > 99.0%), cyanuric acid (Aladdin, >98.0%), nitric acid (Aladdin, AR, 65-68%), boric acid (Rgent, AR > 99.5%), cobalt nitrate (Guangfu, AR > 99.0%), 1-ethyl-3-methylimidazolium tetrafluoroborate (Aladdin, AR > 99.0%), 1-ethyl-3-methylimidazolium bis (trifluoromethylsulfonyl) imide (Aladdin, AR > 99.0%), 1-butyl-3-methylimidazolium tetrafluoroborate (Aladdin, AR > 99.0%), 1-ethyl-3-methylimidazolium hexafluorophosphate (Aladdin, AR > 99.0%), methanol (Fuyu, AR > 99.5%), ethanol (Fuyu, AR > 99.7%).

Synthesis of CoO_x on CN (CoO_x-CN). As-obtained CN (0.2 g) was dispersed in deionized H_2O (100 mL) with vigorous stirring in a bottom flask. The aqueous

$Co(NO_3)_2$ solution with the concentration of 1.5 × 10^{-5} mol L^{-1} (50 mL) was then added into the even CN suspension. The resulting aqueous suspension was stirred at room temperature for 1 h. Finally, the solid powder was centrifuged and washed with absolute ethanol thoroughly, and then dried in the vacuum at 60 °C. The sample was denoted as CoO_x-CN.

Sample characterization

Higher harmonics were eliminated by detuning the double crystal Si (111) monochromator. Three gas-filled ionization chambers were used in series to measure the intensities of the incident beam (Io), the beam fluorescence-emitted by the sample (It), and the beam subsequently transmitted by the reference foil (Ir). The third ion chamber was used in conjunction with the reference metal foil for the elemental edge measurements. All measurements were compared with the reference samples. Control of parameters for Extended X-ray Absorption Fine Structure (EXAFS) measurements, collection modes, processing of data, and calculation of errors was performed following the guidelines set by the Standards and Criteria Committee of the International XAFS Society. The EXAFS function, χ was obtained by subtracting the post-edge background from the overall absorption coefficient and then normalizing to the edge jump. The normalized function, $\chi(E)$ was then converted to k space, which is the photoelectron wave vector. The wave-vector function $\chi(k)$ was weighted by k^3 to compensate for the damping of the backscattering oscillation in the high k region. Subsequently, k^3-weighted $\chi(k)$ data ranging from ca. 2 to ca. 10 $Å^{-1}$ at the elemental edges were converted to r space by Fourier transformation to identify the backscattering contributions of each coordination shell. A nonlinear least-squares algorithm was employed to curve-fitting in the r space ranging from ca. 1 to ca. 2 Å at the elemental edges. All the computer programs were implemented in UWXAFS 3.0 package, and the backscattering amplitude and phase shift of the specific-atom model were calculated by the FEFF8 code. In this analysis, the structural parameters corresponding to first-shell coordination, such as coordination numbers (N), bond length (R), Debye–Waller factor (σj2), and inner potential shift (ΔE_0), have been calculated. The electron paramagnetic resonance (EPR) was measured by ·OH radicals which usually can be trapped by 5,5-dimethyl-l-pyrroline N-oxide (DMPO), producing the EPR signals of their adducts. The sample (5 mg) was dissolved in the DMPO solution with H_2O as a solvent to obtain the liquid mixture. After illumination, the mixture was characterized using a Bruker EMX plus model spectrometer operating at room temperature. Electrochemical impedance spectroscopy (EIS) measurements were performed with Na_2SO_4 (0.5 mol L^{-1}) solution as the electrolyte using an IVIUM V13806 electrochemical workstation with the three-electrode system, over the frequency range from 102 to 105 Hz with an amplitude of 10 mV (Root Mean Square) and a bias of 0.4 V. Atmosphere-controlled surface photovoltage spectroscopy (AC-SPS) measurements were implemented on home-built apparatus, equipped with a lock-in amplifier (SR830, USA) synchronized with a light chopper (SR540, USA) under different atmospheric conditions. The photoluminescence (PL) spectra of the samples were measured with a spectrofluoro-photometer (LS55

Perkin-Elmer) at an excitation wavelength of 300 nm.

Analysis of produced hydroxyl radicals under illumination. The sample (0.05 g) was dispersed in a coumarin aqueous solution (0.001 mol L^{-1}, 50 mL) in a beaker. Prior to irradiation, the reactor was magnetically stirred for 10 min to attain an adsorption-desorption equilibrium in dark. After irradiation for 1 h with a 150 W Xenon lamp (GYZ220 made in China), the sample was centrifuged. And then a certain volume of solution was transferred into a pyrex glass cell for the fluorescence measurement of 7-hydroxycoumarin at 332 nm excitation wavelength with an emission peak wavelength at 460 nm through the spectrofluoro-photometer (LS55 Perkin-Elmer)

Electrochemical (EC) experiments. Electrochemical (EC) measurements were carried out in a traditional three-electrode system with the prepared film electrodes as the working electrode, a platinum plate (99.9%) as the counter electrode, a saturated KCl Ag/AgCl electrode as the reference electrode, and 0.5 mol L^{-1} Na_2SO_4 solution as the electrolyte. EC experiments were performed in a quartz cell with a high-purity N_2 or CO_2 bubbled system. An IVIUM V13806 electrochemical workstation was employed to test EC performance, and all the experiments were performed at room temperature (about 25 °C).

Recycle test. After each run for 3 h, the used photocatalyst was separated, washed with plenty of deionized water and ethanol then dried at 60 °C in the vacuum oven for the next cycle.

The verification of produced H_2O_2. The possible production of H_2O_2 in a tiny amount of water used in the photocatalytic CO_2 reduction was examined by the iodometry method. Specifically, 0.5 ml of 0.4 mol L^{-1} potassium iodide (KI) solution and 0.5 ml of 0.1mol L^{-1} potassium hydrogen phthalate ($C_8H_5KO_4$) solution were added to 1 mL liquid at the bottom of the reaction, cell and kept for 30 min. H_2O_2 would react with I^- under acidic conditions to form I^{3-} ($H_2O_2 + 3I^- + 2H^+ \rightarrow I^{3-} + 2H_2O$), which has a strong absorption at about 350 nm. The content of I^{3-} was determined by measuring the absorbance at 350 nm by UV–vis spectroscopy, from which the total amount of H_2O_2 produced during the reaction can be calculated.

Transient absorption spectroscopy (TAS). Microsecond transient absorption decays were acquired in transmission mode with a home-built setup. A Nd:YAG nanosecond laser (Spectra-Physics, LAB-130-10) was used as the pump light and the 355 nm output light was focused on the sample films. Typical excitation densities of 0.5 mJ cm^{-2} and laser repetition rates of 10 Hz were used. Probe light was generated by a 100 W ASBN-W tungsten-halogen lamp. Long-pass and short-pass filters were positioned between the lamp and the sample to minimize the short wavelength irradiation and heating of the sample, respectively. Absorption changes from the sample were collected and relayed to a monochromator (Spectral Products, CM110) to select the probe wavelength. The time-resolved signal was collected with a Si photodiode. The signal was amplified by a wideband preamplifier and recorded by an oscilloscope (Tektronics DPO4012). Transient absorption decays were typically obtained from an average of 256 laser pulses.

We convert the relative change in the detected light intensity by using transmission mode to the absorbance signal based on the following relation:

$$10^{-\Delta A} = \frac{T_t}{T_0} \quad (1)$$

where ΔA is the change in absorbance (in absorbance units), T is the transmission (in decimal units) of the film, and the subscripts 0 and t indicate either the initial value or the value at time t.

For each time TAS measurement, pure CO_2 was bubbled into deionized water then the resultant gaseous CO_2/H_2O vapour mixture was introduced into the test cell from the headspace for 30 minutes to achieve the equilibrium adsorption of reactant molecules on the surface of the sample film. Besides, for the same procedures, N_2 was employed to replace the CO_2 to obtain TAS data as a reference.

In-situ fourier transform infrared spectroscopy (In-situ FTIR). To detect the reaction intermediates in the CO_2 reduction process, under mimicking conditions with the reaction the in-situ FTIR measurements were performed. The sample was firstly loaded in the sample holder with a flat surface. A cover was fixed on the sample holder to form a reaction cell connected to an evacuation line (∼10−7 mbar). The reaction cell was evacuated to remove all adsorbed impurities and subsequently purged with water vapor-saturated 25% CO_2 gas flow (diluted by N_2). After the equilibrium of CO_2 and water adsorption on the photocatalyst was reached, the FTIR data was collected as background. Subsequently, UV–vis light irradiation was introduced to the reaction space via an observation window and the FTIR data was collected with gas flowing. For isotopic FTIR, water vapor-saturated $^{12}CO_2$ or $^{13}CO_2$ - without any dilution was purged into the reaction cell until reaching the adsorption equilibrium, then the reaction cell was sealed for the subsequent photocatalytic reactions.

Statistical analysis. All the data for photocatalytic activities were presented as means ± standard deviation (SD). In order to test the significance of the observed differences between the study groups, analysis by variance (ANOVA) statistics was applied and a value of $P < 0.05$ was considered to be statistically significant.

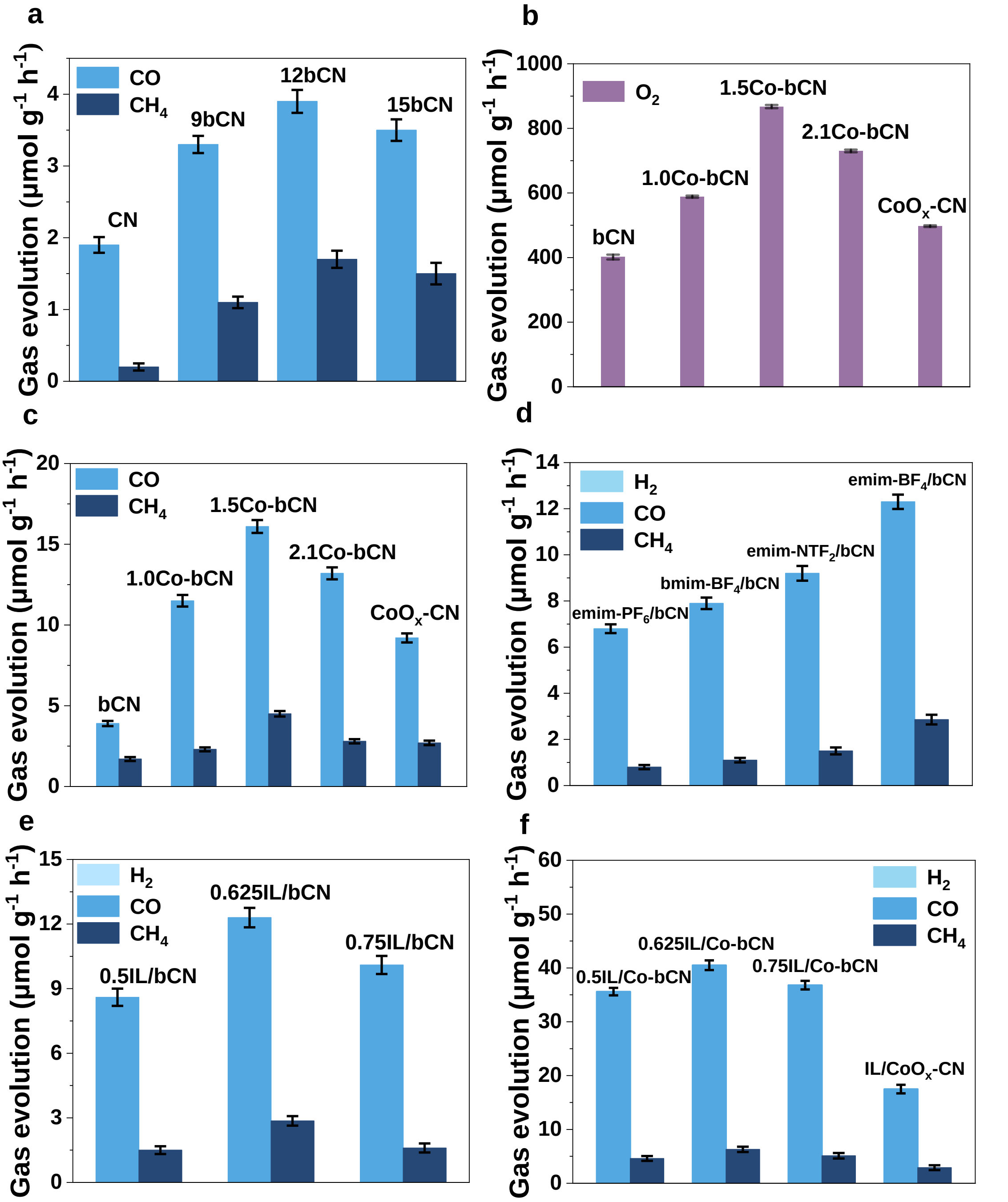

Supplementary Figure 1. Photocatalytic activities of (a) *x*bCN for CO_2 reduction reaction (CO_2RR), (b) *y*Co-bCN for water oxidation using $AgNO_3$ as electron scavenger, (c) *y*Co-bCN, (d) different IL/bCN, (e) *m*IL/bCN and (f) *n*IL/1.5Co-bCN for CO_2RR. Note: *x*% indicates the mass ratio (9, 12 and 15%) of boric acid to CN; *y*% indicates the concentration (1.0, 1.5 and 2.1 × 10^{-5} mol L^{-1}) of $Co(NO_3)_2$ solution used for synthesis of Co single-atom modified samples; *m* and *n* indicate the IL concentration (0.5, 0.625 and 0.75 mmol L^{-1}) used for the synthesis of IL modified samples. Data are presented as the mean ± SD from three independent experiments. The calculation of stoichiometric chemistry of IL/Co-bCN in Figure 1 for photocatalytic CO_2 reduction is as below: Electron number = electron (CO) + electron (CH_4) = 40.5 × 2 + 6.3 × 8 = 131.4; Hole number = hole (O_2) = 33.9 × 4 = 135.6.

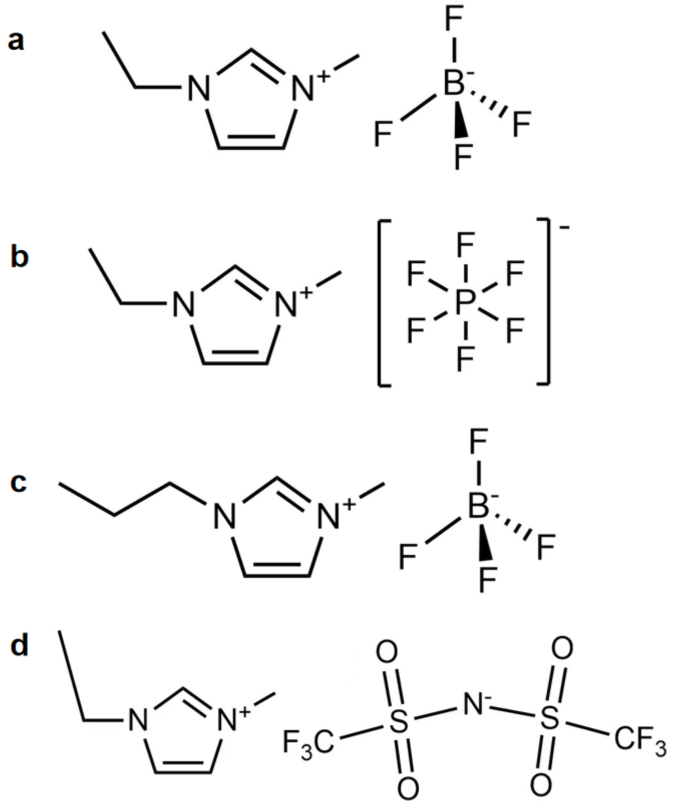

Supplementary Figure 2. The chemical structures of ionic liquids applied in this work. (a) 1-ethyl-3-methylimidazolium tetrafluoroborate ([emim][BF_4]), (b) 1-ethyl-3-methyl imidazolium hexafluorophosphate ([emim][PF_6]), (c) 1-butyl-3-methylimidazolium tetrafluoroborate ([bmim][BF_4]), and (d) 1-ethyl-3-methylimidazolium bis (trifluoromethyl) sulfonylimide ([emim][NTF_2]).

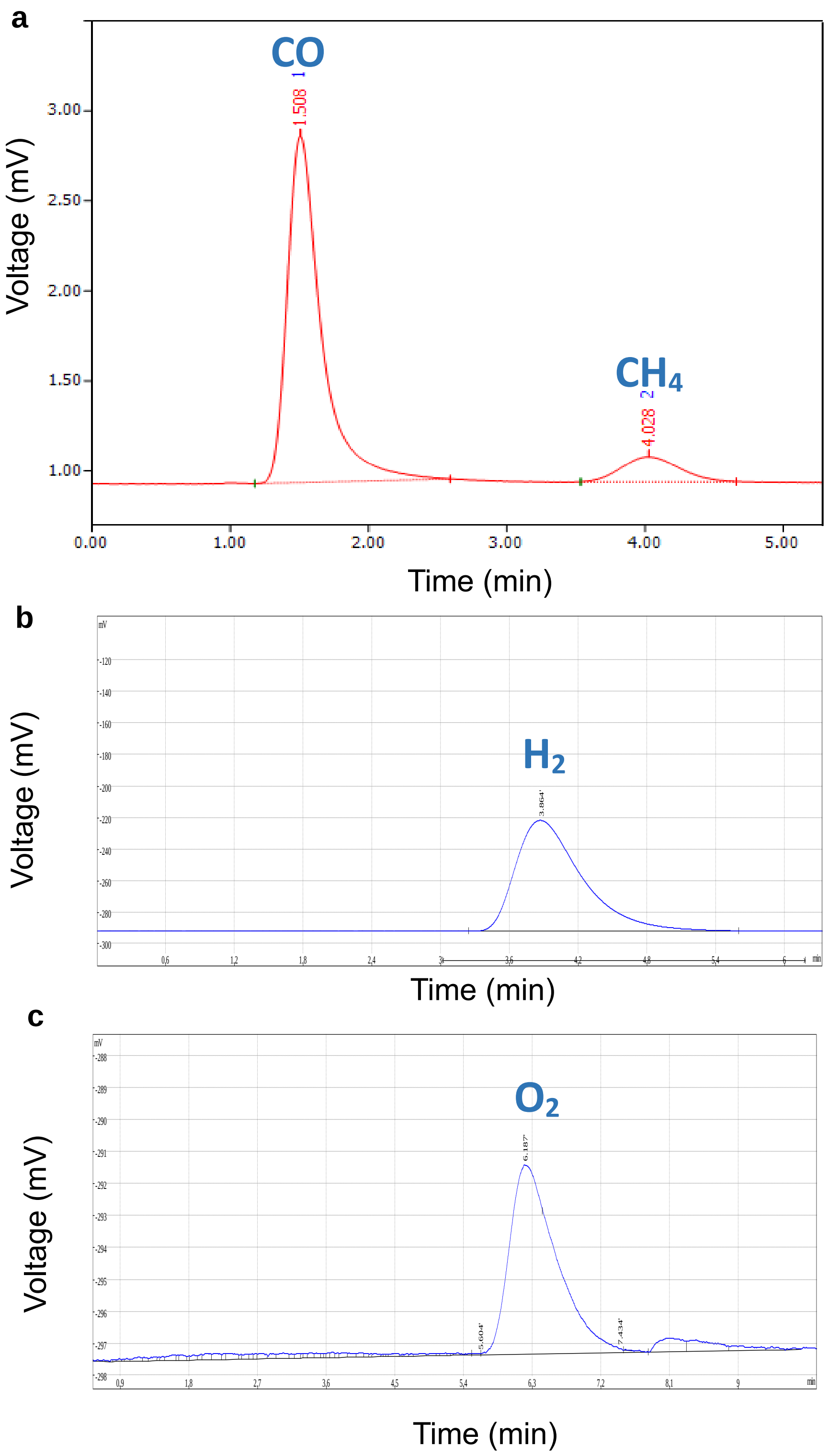

Supplementary Figure 3. (a) GC raw data: gas chromatography spectra (GC-7920, CEAULIGHT with FID detector) of CO and CH_4 as products for photocatalytic CO_2RR. GC raw data: gas chromatography spectra (KE CHUANG GC-2002 with TCD detector) of (b) H_2 and (c) O_2 over IL/Co-bCN for photocatalytic CO_2RR.

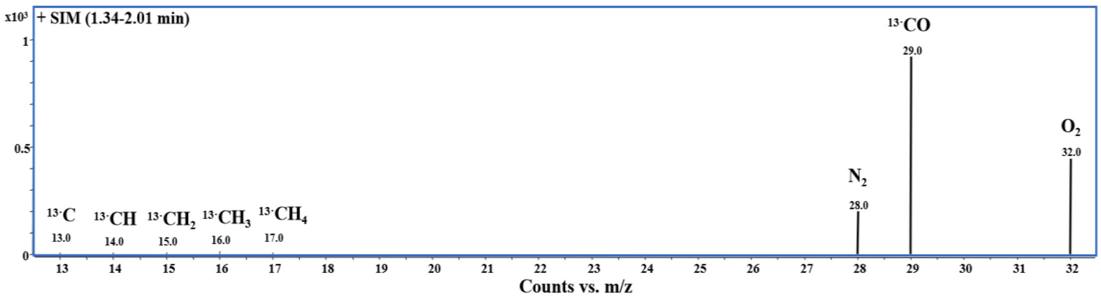

Supplementary Figure 4. GC-MS raw data: selective ion detection chromatography spectra of the products for photocatalytic CO_2RR over IL/Co-bCN with isotopic $^{13}CO_2$ as the reactant.

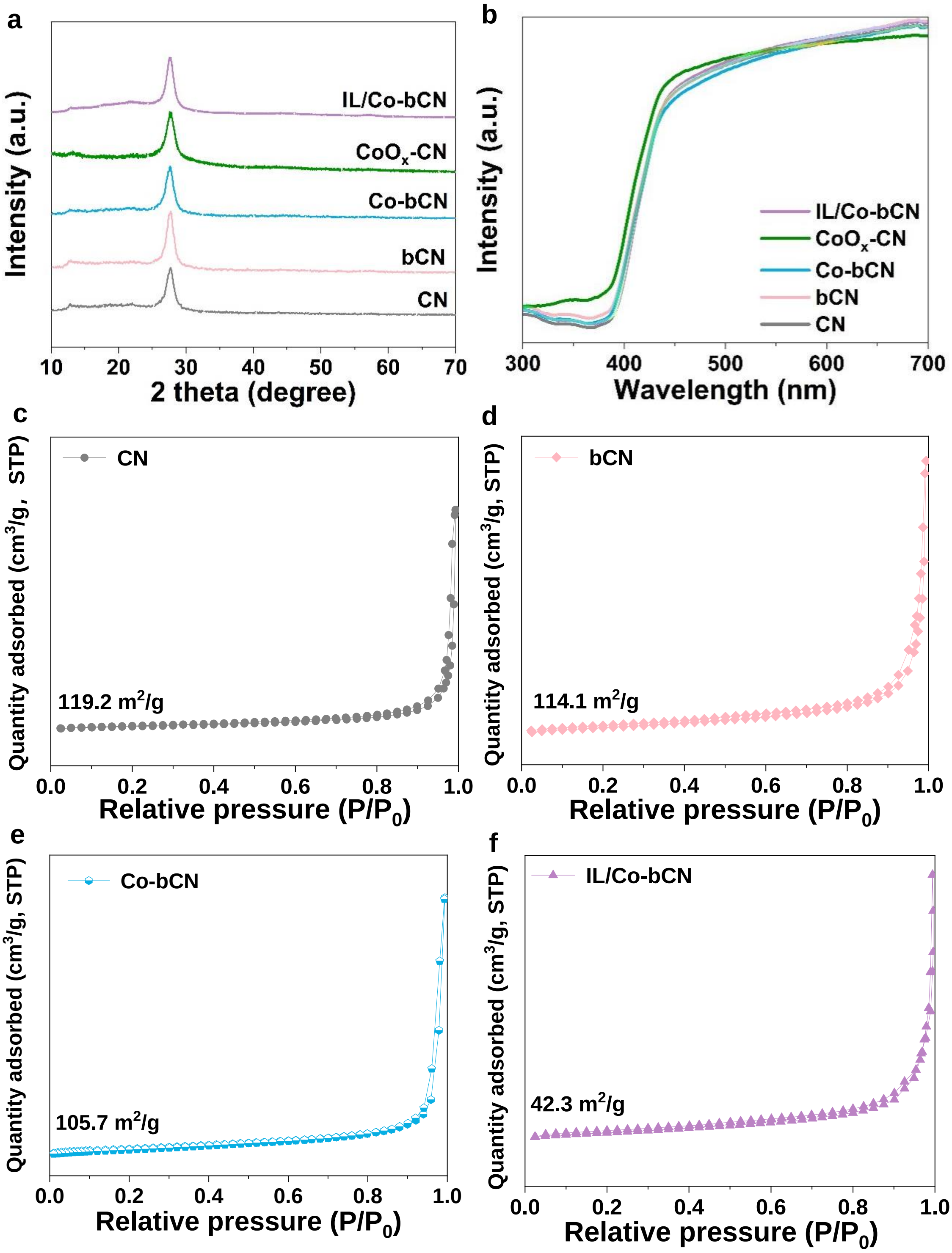

Supplementary Figure 5. (a) XRD patterns and (b) UV–Vis DRS spectra of CN, bCN, Co-bCN, IL/Co-bCN and CoO_x-CN. The N_2 sorption isotherms for (c) CN, (d) bCN, (e) Co-bCN and (f) IL/Co-bCN.

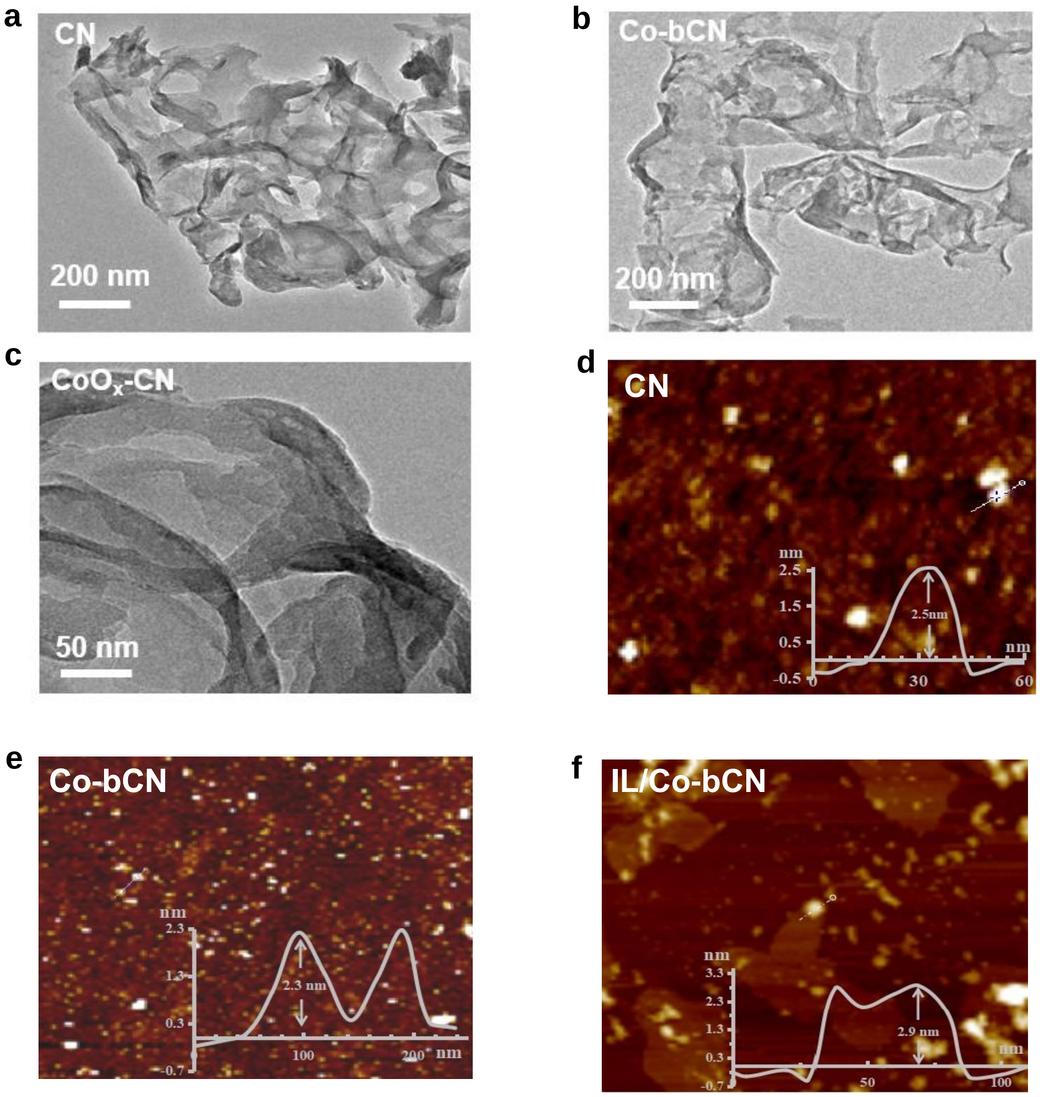

Supplementary Figure 6. TEM images of (a) CN, (b) Co-bCN and (c) CoO_x-CN. AFM images and the corresponding height profiles of (d) CN, (e) Co-bCN and (f) IL/Co-bCN.

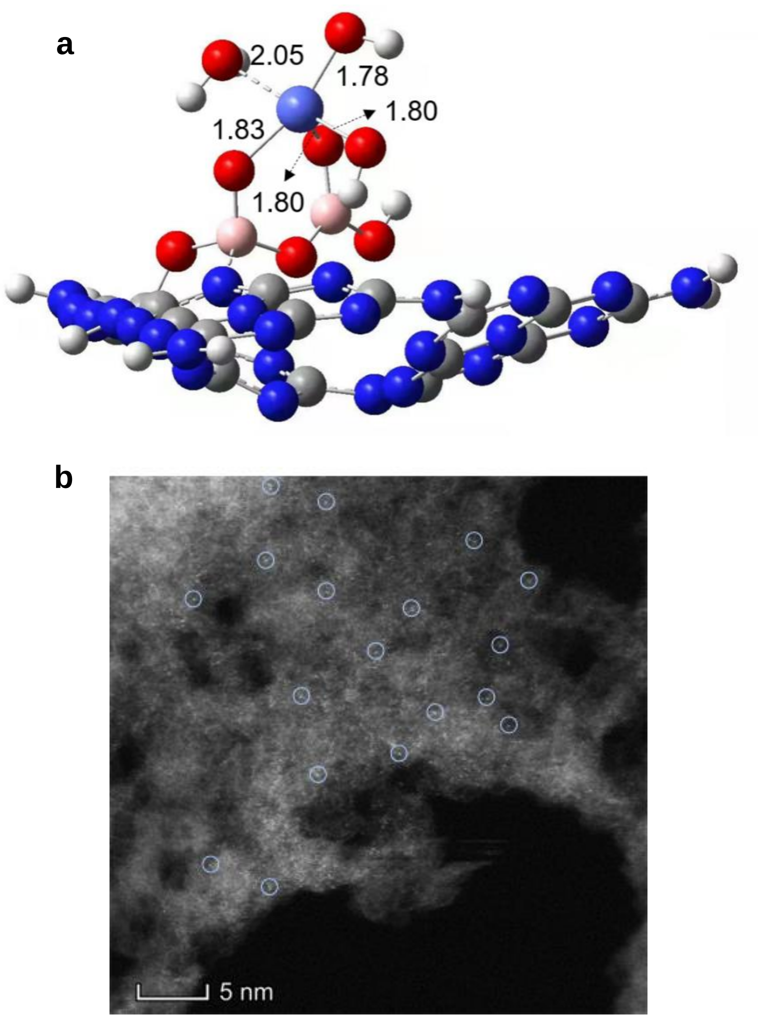

Supplementary Figure 7. (a) Simulated Co single atom with five Co-O bond lengths marked (the unit of bond length is Å). (b) HAADF-STEM image of IL/Co-bCN. Dots in blue circles indicate Co single atoms. Scale bar: 5 nm.

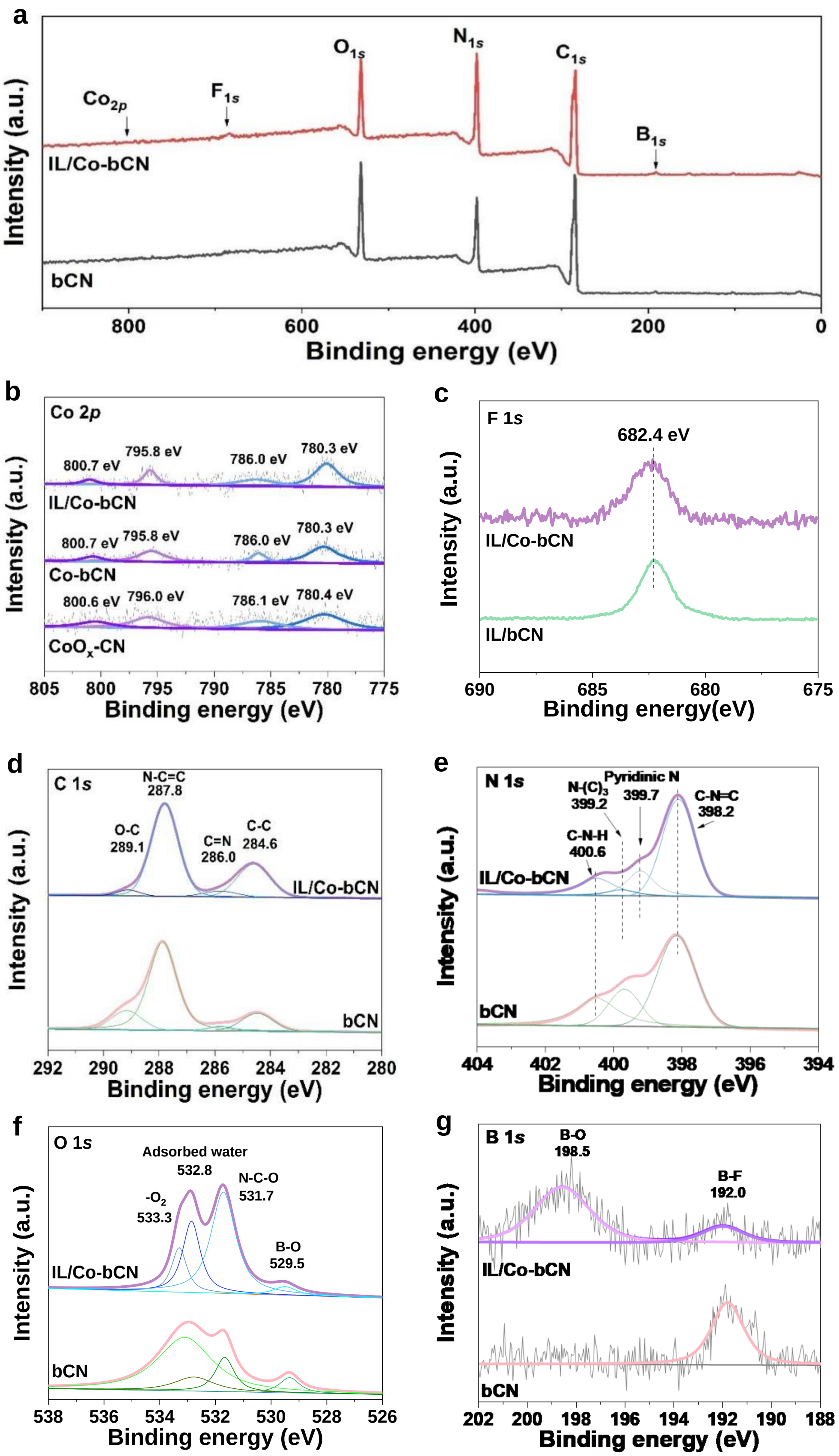

Supplementary Figure 8. XPS spectra of (a) IL/Co-bCN, (b) Co 2*p* of Co-bCN, IL/Co-bCN and CoO_x-CN. (c) F 1*s* of IL/bCN and IL/Co-bCN, (d) C 1*s*, (e) N 1*s*, (f) O 1*s* and (g) B 1*s* of bCN and IL/Co-bCN, respectively.

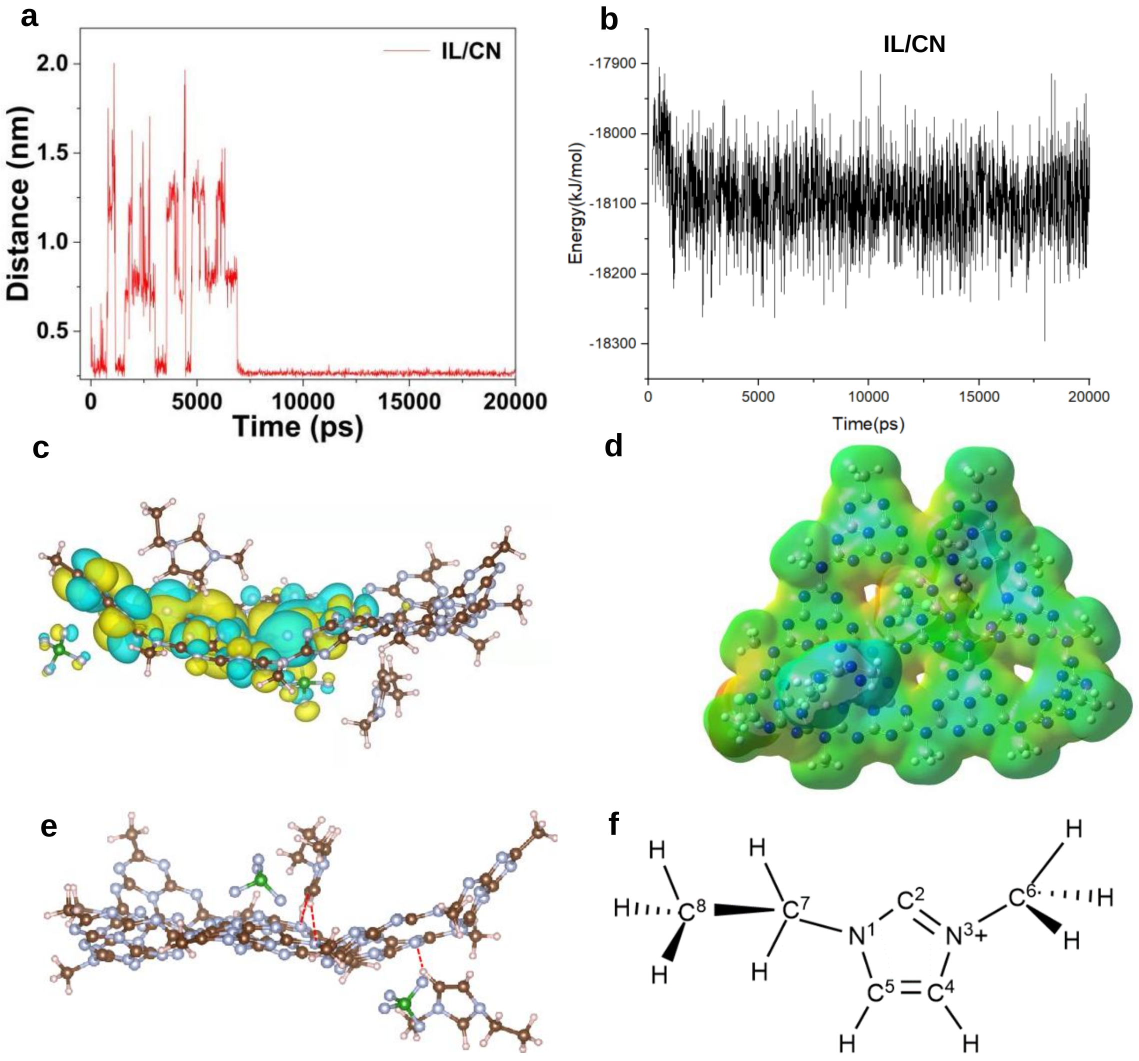

Supplementary Figure 9. (a) The mass center equilibrium distance from the centroid of IL to CN when IL is loaded on the CN surface according to the 20000 ps MD simulation results. (b) The energy result of MD simulation for IL/CN. (c) The electronic structure of equilibrium geometry of IL/CN. (d) The electrostatic potential of IL/CN. (e) The hydrogen bond characteristics of IL/CN interface obtained by MD simulations. f) The illustration of 1-ethyl-3-methylimidazolium cation ($[emim]^+$) with numbered C and N atoms.

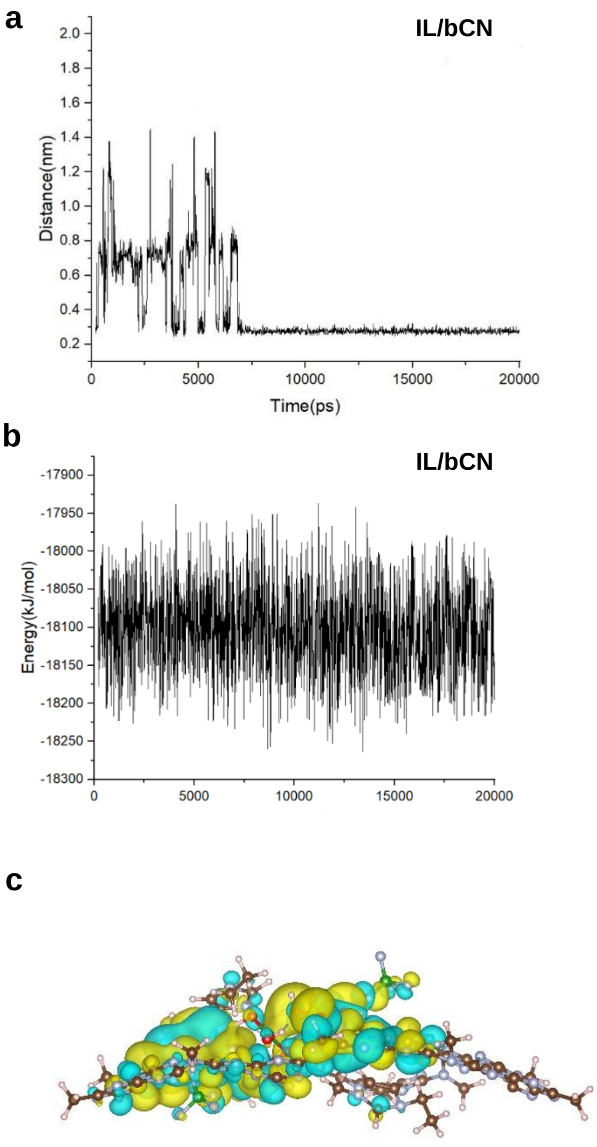

Supplementary Figure 10. (a) The mass center equilibrium distance from the centroid of IL to bCN when IL is loaded on the bCN surface according to the 20000 ps MD simulation results. (b) The energy result of molecular dynamics simulation for IL/bCN. (c) The electronic structure of equilibrium geometry of IL/bCN.

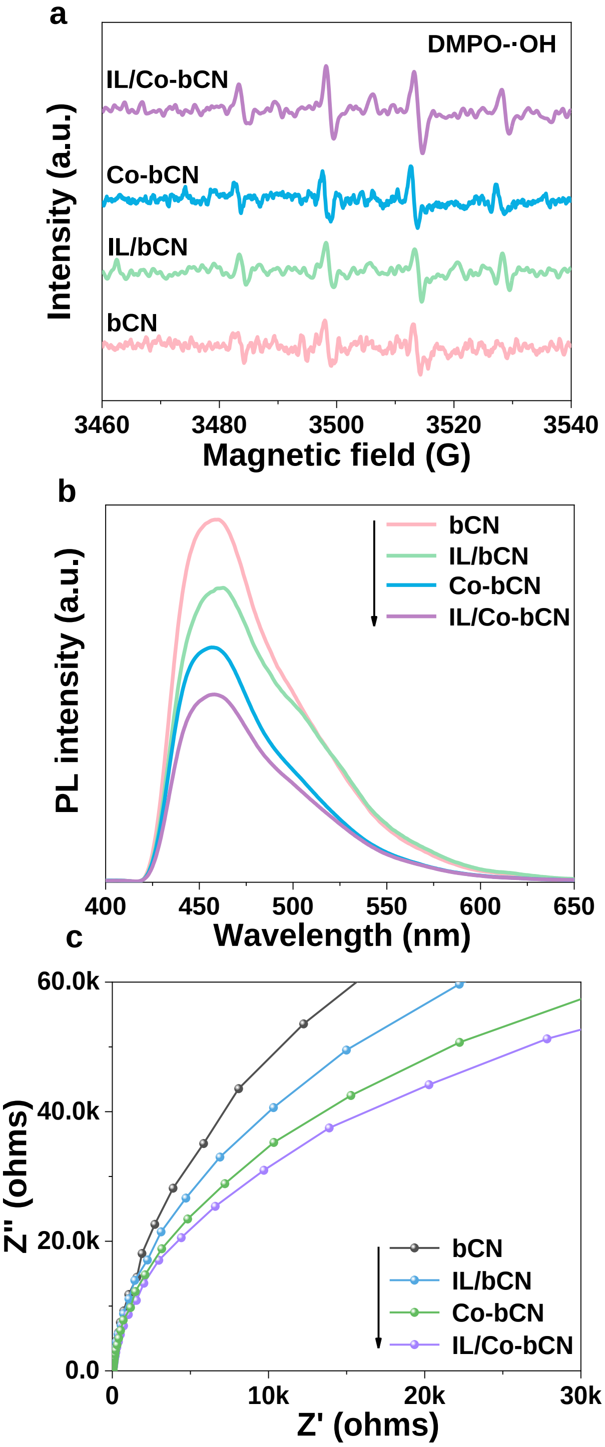

Supplementary Figure 11. (a) EPR spectra of DMPO-·OH adducts, (b) PL and (c) EIS spectra of bCN, IL/bCN, Co-bCN and IL/Co-bCN, respectively.

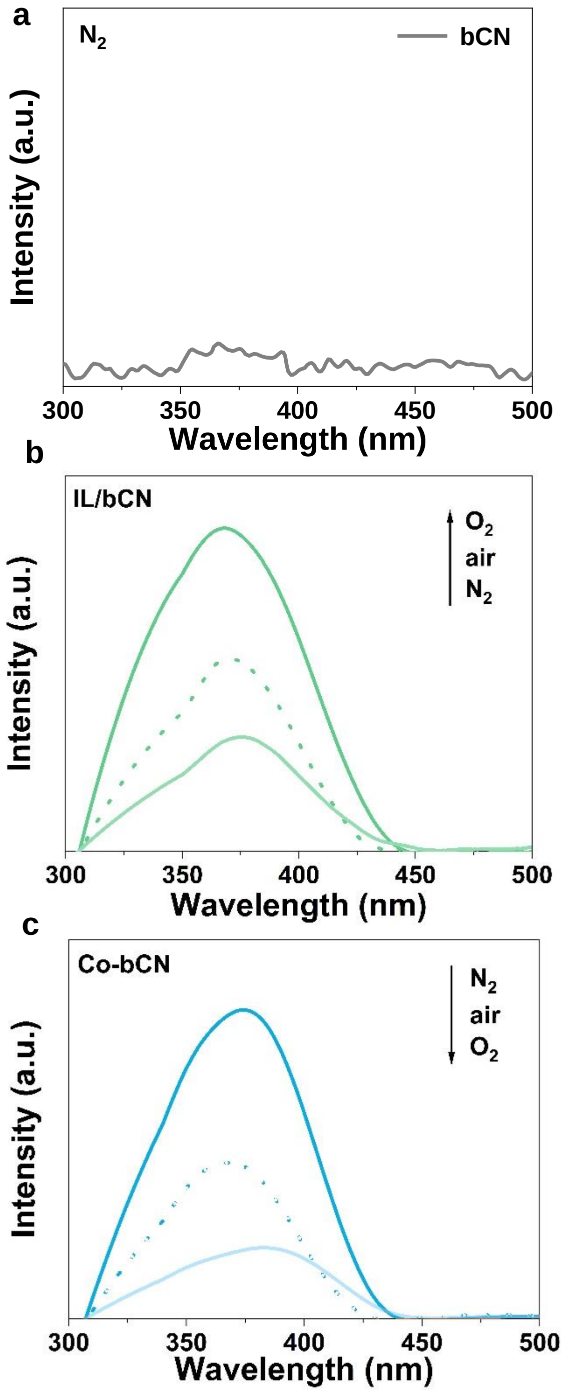

Supplementary Figure 12. AC-SPS responses of (a) bCN, (b) IL/bCN and (c) Co-bCN in different atmospheres.

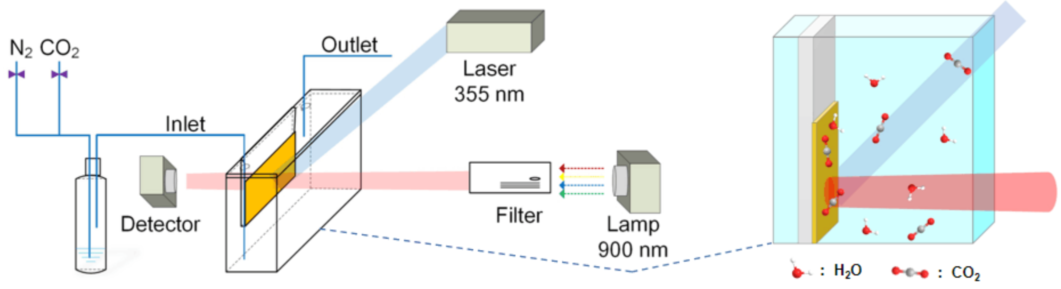

Supplementary Figure 13. Schematic illustration of the TAS setup. For in-situ TAS measurement, the gas was introduced into the reactor cell from the headspace.

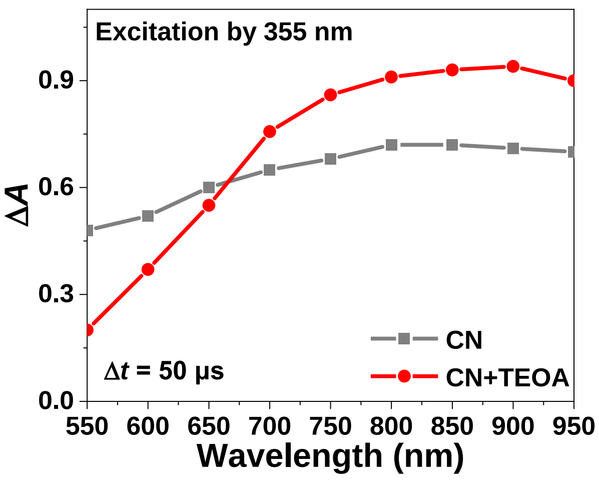

Supplementary Figure 14. Wavelength-dependent transient absorption of CN films in H_2O (gray square) and 10 mmol L^{-1} aqueous triethanolamine (TEOA) solution (red dot).

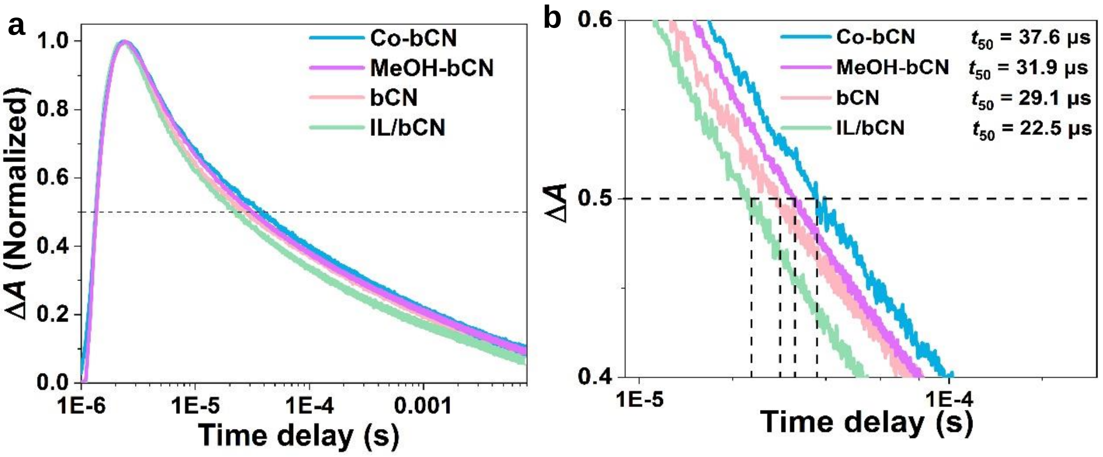

Supplementary Figure 15. (a) TAS decay kinetics of the bCN, IL/bCN, Co-bCN and methanol (MeOH)-bCN in the gaseous N_2/H_2O vapour mixture excited by pulsed 355 nm and monitored at 900 nm. To measure the TAS kinetics of MeOH-bCN, N_2 was bubbled into the aqueous MeOH solution (10 mmol L^{-1}) to carry MeOH in the test cell. (b) The enlarged view of the TAS kinetics to show the lifetimes.

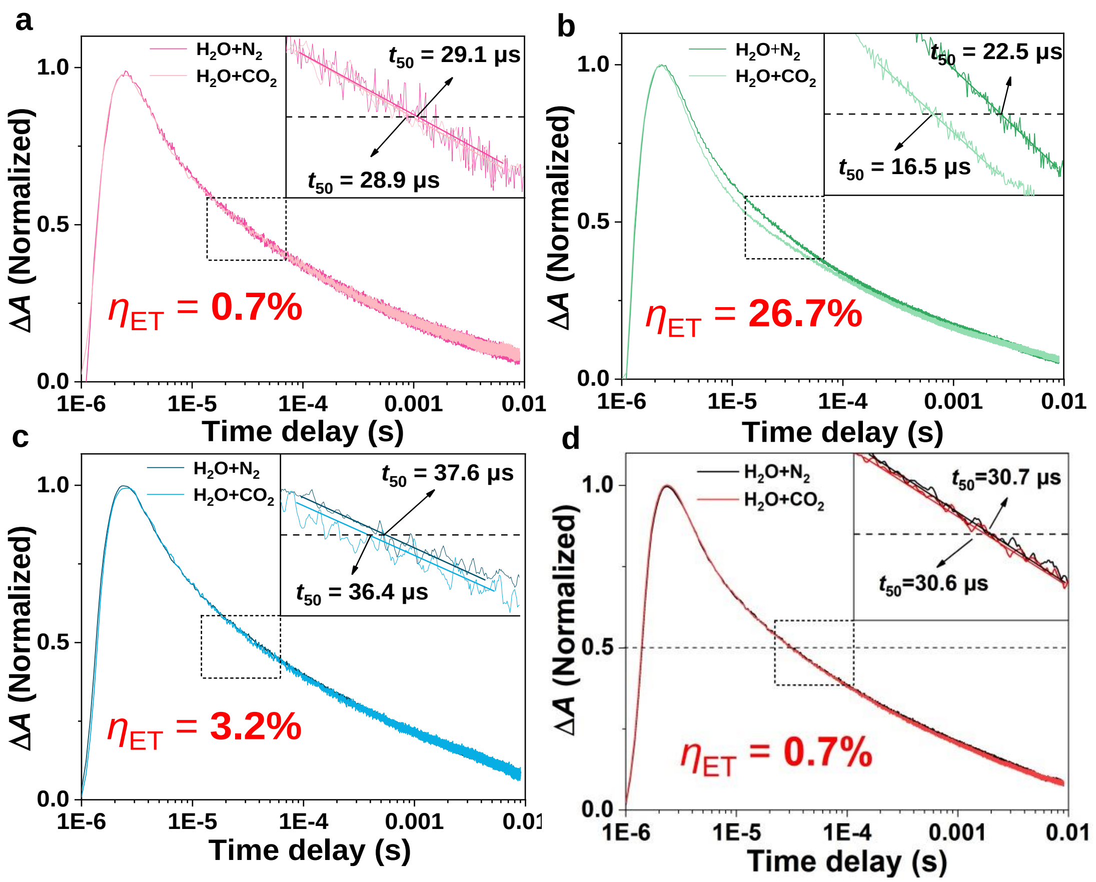

Supplementary Figure 16. TAS decay kinetics of (a) bCN, (b) IL/bCN, (c) Co-bCN and (d) CN in the presence of different gaseous environments excited by 355 nm pulsed laser and monitored at 900 nm.

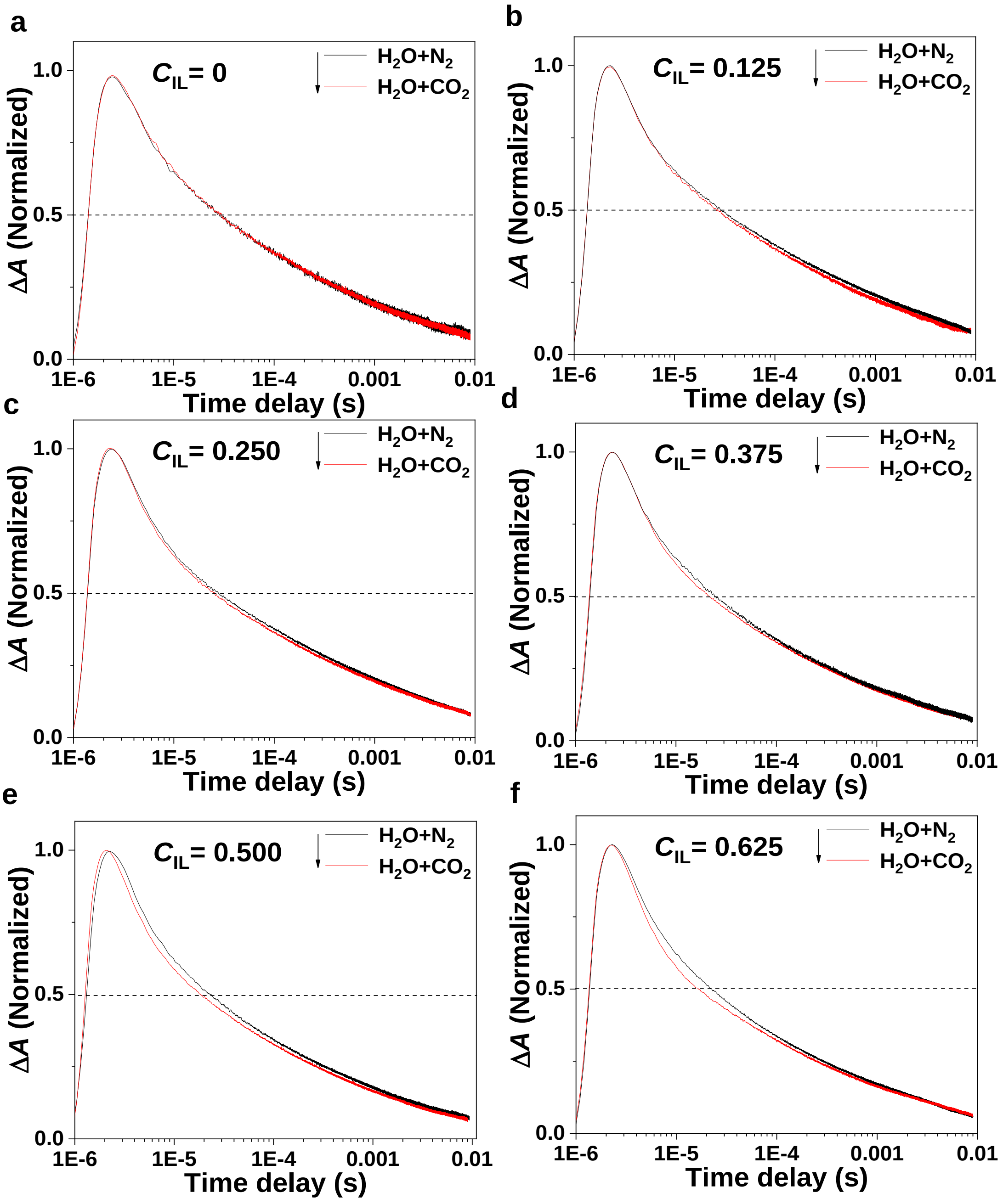

Supplementary Figure 17. TAS decay kinetics of IL/bCN samples in the presence of various IL concentrations of (a) 0, (b) 0.125, (c) 0.250, (d) 0.375, (e) 0.500 and (f) 0.625 mmol L^{-1} modified on bCN samples. The TAS curves were monitored at 900 nm and excited by 355 nm pulsed laser.

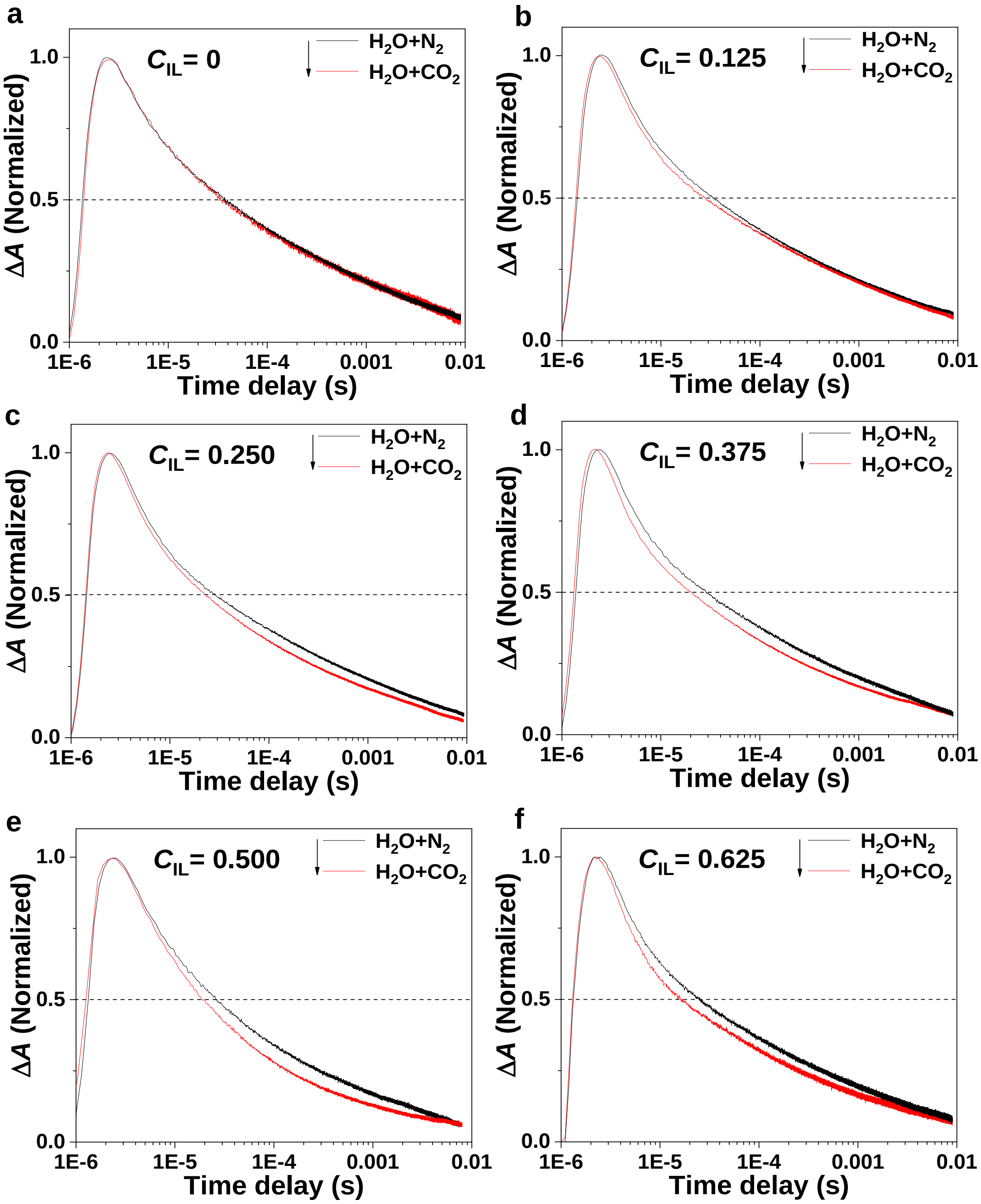

Supplementary Figure 18. TAS decay kinetics of IL/Co-bCN samples in the presence of various IL concentrations of (a) 0, (b) 0.125, (c) 0.250, (d) 0.375, (e) 0.500 and (f) 0.625 mmol L^{-1} modified on Co-bCN samples. The TAS curves were monitored at 900 nm and excited by 355 nm pulsed laser.

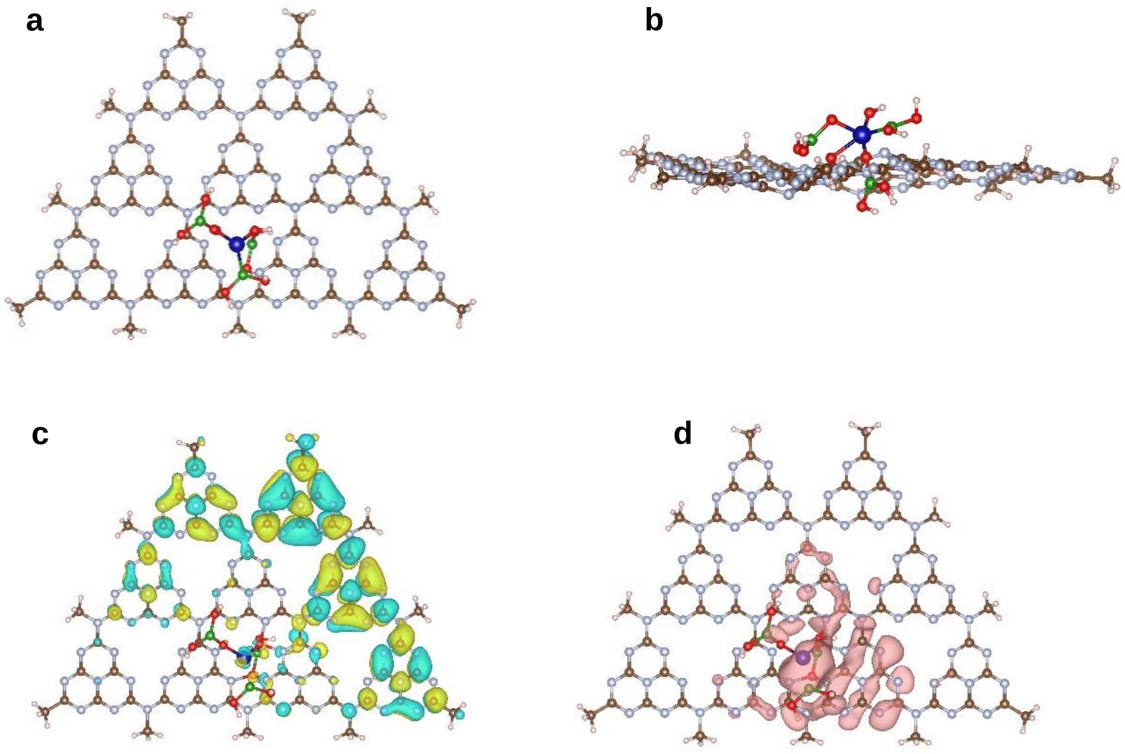

Supplementary Figure 19. (a) Simulated Co-bCN system by density functional theory method in (a) top view and (b) side view, respectively. (c) Differential charge density diagram and (d) hole distribution of Co-bCN.

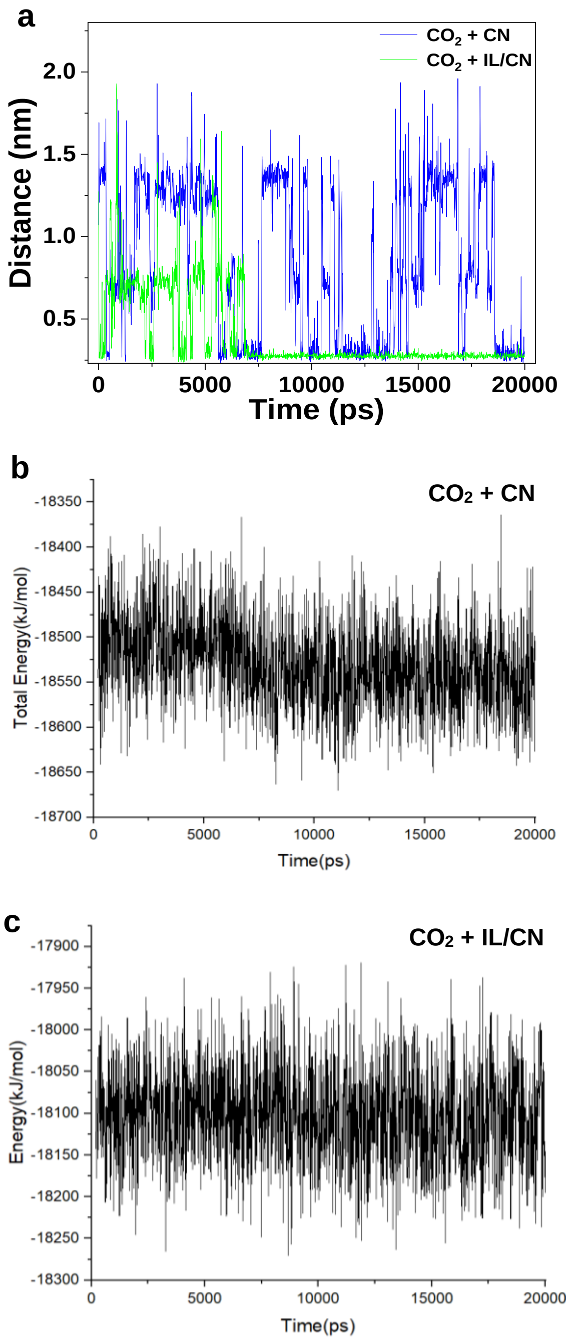

Supplementary Figure 20. (a) The mass center equilibrium distance between IL, CO_2, and CN according to the 20000 ps molecular dynamics simulation results. The blue line indicates the distance between CO_2 and CN when the CO_2+CN system is stable, while the green line indicates the distance from the centroid of CO_2 to CN when the CO_2 + IL/CN system is stable. The energy results of molecular dynamics simulation for (b) CO_2 + CN and (c) CO_2 + IL/CN systems, respectively.

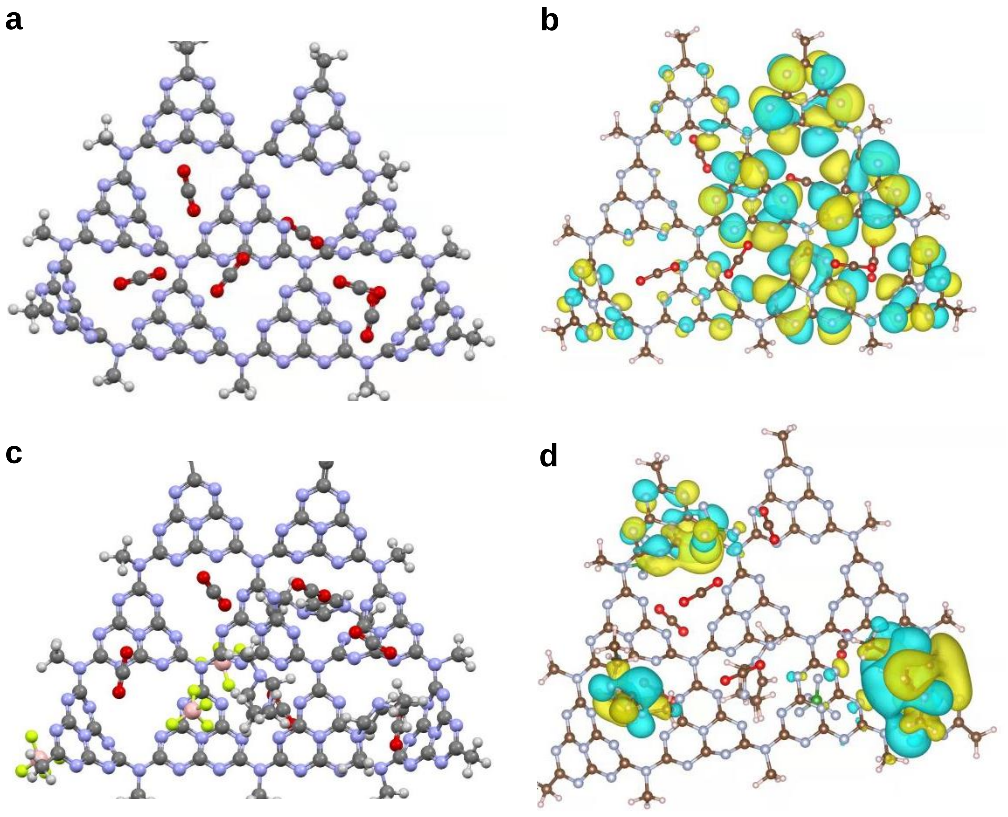

Supplementary Figure 21. (a) The equilibrium geometry of CO_2 adsorption on CN. (b) Differential charge density diagram of CO_2 on CN. (c) The equilibrium geometry of CO_2 adsorption on IL/CN. (d) Differential charge density diagram of CO_2 on IL/CN.

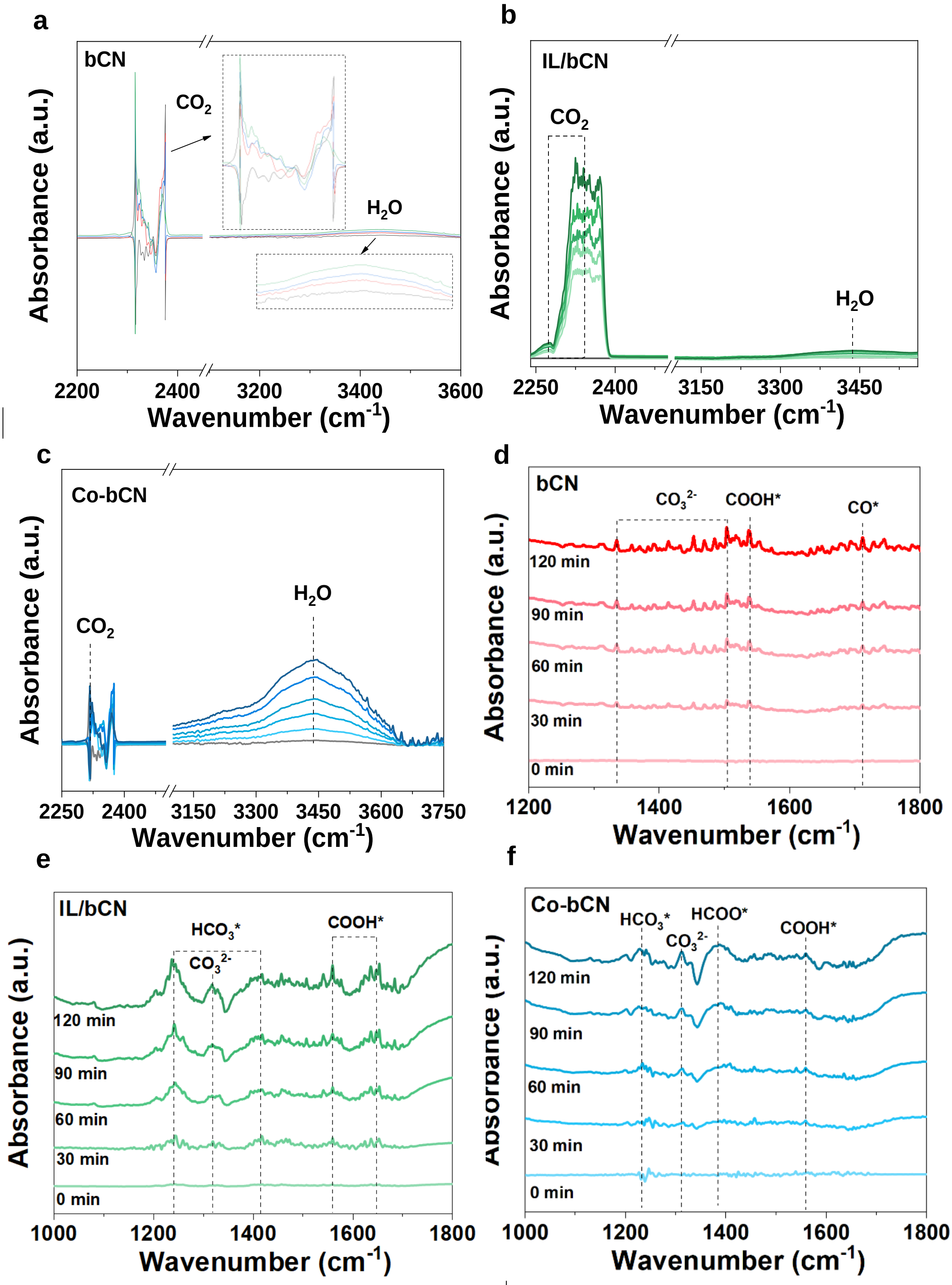

Supplementary Figure 22. FTIR spectra for adsorption of gaseous CO_2/H_2O vapour mixture for (a) bCN, (b) IL/bCN and (c) Co-bCN. In-situ FTIR spectra for the solar-driven CO_2 reduction process on (d) bCN, (e) IL/bCN and (f) Co-bCN, respectively.

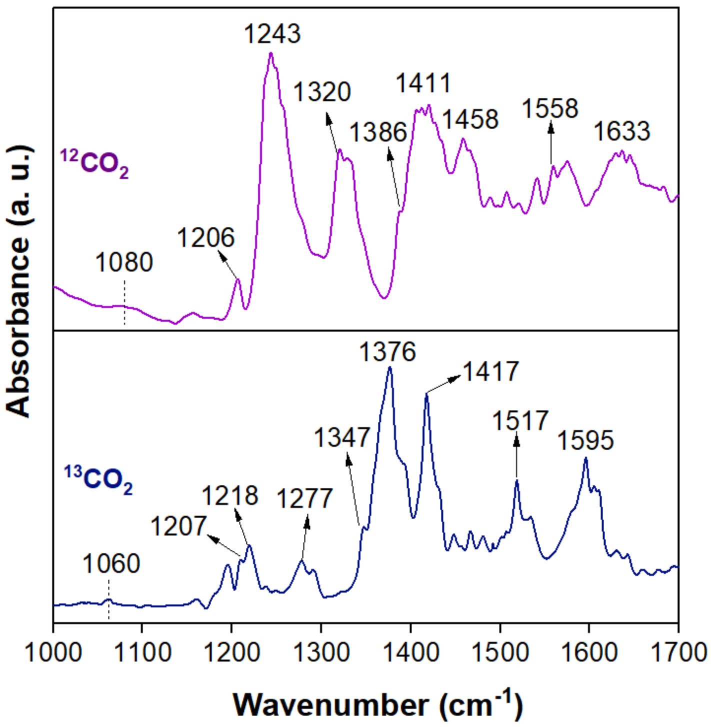

Supplementary Figure 23. In-situ FTIR spectra of the photocatalytic $^{12}CO_2$ (purple) and $^{13}CO_2$ (blue) reduction on IL/Co-bCN, respectively, which were recorded after the illumination for 30 min.

The peak at 1243 cm^{-1} (HCO_3*) in $^{12}CO_2$ FTIR spectra shifts to 1218 cm^{-1} when replacing $^{12}CO_2$ with $^{13}CO_2$. The shift value conforms to the Hooke's law (the error is less than 3 cm^{-1}),[8-10] proving the peaks originate from CO_2. Besides, the peak intensity difference of these two peaks might be attributed to isotopic effect. It is noted that the peaks at 1206 cm^{-1} in $^{12}CO_2$ FTIR spectra and 1207 cm^{-1} in $^{13}CO_2$ FTIR spectra were observed. These nearly identical peaks are not attributed to CO_2-relevant intermediates but possibly peaks due to background or water by-products.[10] Moreover, for the peaks at 1080 cm^{-1} (CHO*), 1320 cm^{-1} (CO_3^{2-}), 1386 cm^{-1} (CO_3^{2-}), 1411 cm^{-1} (HCO_3*), 1458 cm^{-1} (HCO_3*), 1558 cm^{-1} (COOH*) and 1633 cm^{-1} (COOH*), their ^{13}C-counterpart products peaks at 1060 cm^{-1}, 1277 cm^{-1}, 1347 cm^{-1}, 1376 cm^{-1}, 1417 cm^{-1}, 1517 cm^{-1} and 1595 cm^{-1} were detected.[9-10]

Supplementary Table 1. A literature review of g-C_3N_4 based photocatalysts for CO_2 reduction under UV–vis light irradiation.

No.	Photocatalyst	Experimental conditions[a]	Additive or organic solvent	Dual modulation	^{13}C isotopic	O_2 gas measurement	Apparent quantum yield[b] (%)	Production rate (μmol g^{-1} h^{-1})		Turn over frequency[c] (g^{-1} h^{-1})
								CO	CH_4	
1	IL/Co-bCN	Moist H_2O, 25 mg cat, RT, 300W Xe lamp, M_C = 0.125 mmol	×	√	√	√	1.00 (405 nm)	40.5	6.3	0.3740
2	Cr,B-g-C_3N_4[11]	Moist CO_2, 25 mg cat, RT, 500W Xe lamp, M_c = 3.4 mmol	×	×	×	×	-	11.64	0.38	0.0350
3	LDH/RGO/CN[12]	0.4 mL H_2O, 50 mg cat, RT, 300W Xe lamp, M_c = 2.6 mmol	×	×	√	√	0.45 (385 nm)	10.11	0.5	0.0040
4	g-C_3N_4-N-TiO_2[13]	Moist CO_2, 100 mg cat, RT, 300W Xe lamp M_c = 2.6 mmol	×	×	×	×	-	1.23	0.32	0.0006
5	CuO_x-MnO_x/g-C_3N_4[14]	5 mL H_2O, 100mg ca, RT, 300W Xe lamp, M_c: 2.0 mmol	×	√	×	√	-	2.30	0.30	0.0013
6	$W(CO)_6$-CCN[15]	Moist CO_2, 5 mg cat, RT, 300W Xe lamp, M_c = 3.2 mmol	×	×	√	×	5.1 (350 nm)	5.75	4.45	0.0031
7	O-La-CN[16]	4 mL H_2O, 6 mL MeCN, 2 mL TEOA CO_2, 10mg cat, RT, 300W Xe lamp, M_c = 7.2 mmol	√	×	×	×	-	92	5.6	0.0136
8	Coqpy@mpg-C_3N_4[17]	0.05 mol BIH, 0.03 mol PhOH CO_2, 6mg cat, RT, 100W Xe lamp,	√	×	√	×	0.25 (400 nm)	1.15	0.06	0.0004

		M_c = 3 mmol								
9	Co-rGO/C_3N_4[18]	6 mL H_2O, 18 mL acetonitrile, and 6 mL triethanolamine CO_2, 20mg cat, RT, 300W Xe lamp, M_c = 1.2 mmol	√	×	√	×	0.7 (435 nm)	0.55	0	0.0005
10	$Ti_3C_2(OH)_2$/g-C_3N_4[19]	Moist CO_2, 40mg cat, RT, 300W Xe lamp, M_c = 0.2 mmol	×	×	×	×	0.01 (420 nm)	2.24	0.04	0.0110
11	MoO_2/g-C_3N_4[20]	20 mL H_2O CO_2, 50 mg cat, RT, 300W Xe lamp, M_c = 1.1 mmol	×	×	×	×	-	1.98	0.46	0.0020
12	Cu_2O/QDg-C_3N_4[21]	10mL H_2O CO_2, 50 mg cat, RT, 300W Xe lamp, M_c = 0.9 mmol	×	×	×	×	-	8.18	0.08	0.0091
13	Cu-CCN[22]	0.5mL H_2O, 1 mL ethanol CO_2, 25 mg cat, RT, 300W Xe lamp, M_c = 6.6 mmol	√	×	×	×	-	3.09	0.1	0.0005
14	g-C_3N_4@hm-C(CN)$_3$[23]	0.4mL H_2O CO_2, 0.1 g cat, RT, 300W Xe lamp, M_c = 12 mmol	×	×	√	√	0.81 (385 nm)	16.5	0.3	0.0014
15	WP-NC/g-C_3N_4[24]	500 μL H_2O, 20 mL acetonitrile CO_2, 5 mg cat, RT, 300W Xe lamp, M_c = 1.1 mmol	√	×	×	×	-	376	0	0.3418
16	$Cu_{2-x}S$/g-C_3N_4[25]	Moist CO_2, 30 mg cat, RT, 300W Xe lamp, M_c = 1.95 mmol	×	×	√	×	-	319.4	23.7	0.1759

Note: (a) For the description of experimental conditions, RT is short for room temperature; cat is short for photocatalysts. To calculate the turnover frequency (TOF) values, the moles of cocatalyst per gram of photocatalyst, denoted as M_c for short, are calculated based on the cocatalyst amount specified in each work. (b) The defination of apparent quantum efficiency (AQY) is

described as the equation: AQY (%) = $N_{electron}/N_{photon}$ = {[2 × N (CO) + 8 × N (CH_4)] × N_A}/N_{photon}, where the N_A is the Avogadro constant and N (CO) and N (CH_4) are the yield of the evolved CO and CH_4. The calculation of $N_{electron}$ is based on the fact that two electrons are required to produce one molecule CO and eight electrons are required to produce one molecule CH_4. The N_{photon} is calculated using the equation: N_{photon} = [Light intensity × Illumination area × Time] / Average single photon energy. The average single photon energy (E_{photon}) is figured out using the equation: E_{photon} = hc/λ, where h is the Planck constant, c indicates speed of light, and λ is the wavelength. The incident light intensity was measured by a photometer (Mei Kong, TN99D). The total number of incident photons at 405 nm (N_{photon}) was estimated to be 8.33 × 10^{19} photon h^{-1}. (c) Turn over frequency (TOF) is calculated using the equation: TOF = Moles of converted CO_2/(Moles of reduction cocatalyst sites × Reaction time). Noteworthily, taking LINE 7 for example, although the CO production rate is 92 μmol g^{-1} h^{-1}, the TOF value is only 0.0136 h^{-1}.

Supplementary Table 2. EXAFS data fitting results of cobalt in Co-bCN.

Sample	Path	N	ΔE(eV)	100 × R(Å)	10^3 × σ^2($Å^2$)	R-factor
Co foil	Co-Co	12	7.78(0.51)	249.3(0.3)	5.63 (0.41)	0.002
Co-bCN	Co-O	4.96(0.79)	1.75(0.02)	205.1(1.4)	9.88 (2.32)	0.009

Supplementary Table 3. Surface compositions of C, N, O, B, F and Co from XPS results for bCN, Co-bCN and IL/Co-bCN.

Sample	C (at%)	C (wt%)	N (at%)	N (wt%)	O (at%)	O (wt%)	B (at%)	B (wt%)	F (at%)	F (wt%)	Co (at%)	Co (wt%)
bCN	66.12	61.17	16.97	18.38	14.75	18.24	2.16	2.21	0.00	0.00	0.00	0.00
Co-bCN	61.05	57.04	17.26	18.26	19.52	22.44	2.05	2.09	0.00	0.00	0.081	0.15
IL/Co-bCN	61.09	58.14	23.68	23.59	12.35	15.12	1.98	2.06	0.82	0.94	0.080	0.15

Supplementary Table 4. Comparison of dynamics and efficiency for *x*IL/bCN samples.

IL concentration (mmol L^{-1})	t_{50} (μs)	$t_{50(CO_2)}$ (μs)	k_{ET} (s^{-1})	η (100%)
0.000	29.1	28.9	238	0.007
0.125	28.8	26.1	3591	0.093
0.250	27.6	23.3	6687	0.156
0.375	25.5	20.4	9803	0.200
0.500	23.9	18.2	13104	0.238
0.625	22.5	16.5	16161	0.267

Supplementary Table 5. Comparison of dynamics and efficiency for *x*IL/Co-bCN samples.

Concentration of IL （mmol L^{-1}）	t_{50} (μs)	$t_{50(CO_2)}$ (μs)	k_{ET} (s^{-1})	η (100%)
0.000	37.6	36.4	877	0.031
0.125	33.1	27.8	5760	0.160
0.250	29.4	23.4	8721	0.204
0.375	28.7	20.6	13700	0.282
0.500	26.1	18.0	17241	0.310
0.625	25.2	16.3	21667	0.353

Supplementary Reference

1 (a) Adamo, C. Toward reliable density functional methods without adjustable parameters: The PBE0 model. *J. Chem. Phys.* **110**, 6158 (1999); (b) Perdew, J. P. Burke, K. & Ernzerhof, M. Generalized gradient approximation made simple. *Phys. Rev. Lett.* **77**, 3865 (1996); (c) Weigend, F. & Ahlrichs, R. Balanced basis sets of split valence, triple zeta valence and quadruple zeta valence quality for H to Rn: Design and assessment of accuracy. *Phys. Chem. Chem. Phys.* **7**, 3297-3305 (2005).

2 Andrade, J. D. Böes, E. S. & Stassen, H. *J. Phys. Chem. B* **106**, 13344-13351 (2002).

3 Jorgensen, W. Maxwell, D. & Tirado-Rives, J. *J. Am. Chem. Soc.* **118**, 11225-11236 (1996).

4 Martinez, L. Andrade, R. Birgin, E. G. & Martínez, J. M. PACKMOL: A package for building initial configurations for molecular dynamics simulations. *J. Comput. Chem.* **30**, 2157-2164 (2009).

5 Abraham, M. J. van der Spoel, D. Lindahl, E. Hess, B. and the GROMACS development team, GROMACS User Manual version 5.1.4, www.gromacs.org.

6 Berendsen, H. J. Postma, J. P. M. van Gunsteren, W. F. DiNola, A. & Haak, J. R. Molecular dynamics with coupling to an external bath. *J. Chem. Phy.* **81**, 3684-3690 (1984).

7 (a) Grimme, S. Semiempirical GGA-type density functional constructed with a long-range dispersion correction. *J. Comput. Chem.* **27**, 1787 (2006). (b) Grimme, S. Antony, J. Ehrlich, S. & Krieg, H. A consistent and accurate ab initio parametrization of density functional dispersion correction (DFT-D) for the 94 elements H-Pu. *J. Chem. Phys.* **132**, 154104 (2010).

8 (a) Meyers, R. A. et al. Microrheology of biological specimens. *Encyclopedia of Analytical Chemistry.* **1**, 14344 (2001). (b) Lei, K. et al. A Metal-Free Donor–Acceptor Covalent Organic Framework Photocatalyst for Visible-Light-Driven Reduction of CO_2 with H_2O. *ChemSusChem* **13**, 1725 (2020).

9 (a) Hua, S. Hwan Oh, M. Osowiecki, W. Wooyul Kim, T. Paul Alivisatos, A. & Frei, H. Carbon dioxide dimer radical anion as surface intermediate of photoinduced CO_2 reduction at aqueous Cu and CdSe nanoparticle catalysts by Rapid-Scan FT-IR spectroscopy. *J. Am. Chem. Soc.* **140**, 4363-4371 (2018). (b) Di, T. Zhu, B. Cheng, B. Yu, J. & Xu, J. A direct Z-scheme g-C_3N_4/SnS_2 photocatalyst with superior visible-light CO_2 reduction performance. *J Catal* **352**, 532 (2017).

10 (a) Wei, W. et al. Photocatalytic C–C coupling from carbon dioxide reduction on copper oxide with mixed-valence Copper(I)/Copper(II). *J. Am. Chem. Soc.* **143**, 2984-2993 (2021). (b) Tan, H. et al. Unlocking the potential of the formate pathway in the photo-assisted Sabatier reaction. *Nat. Catal.* **3**, 1034 (2020).

11 Ojha, N. Bajpai, A. & Kumar, S. Enhanced and selective photocatalytic reduction of CO_2 by H_2O over strategically doped Fe and Cr into porous boron carbon

nitride. *Catal. Sci. Technol.* **10**, 2663 (2020). Journal of Catalysis 352 (2017) 532–541

12 Yang, Y. et al. Urchin-like hierarchical CoZnAl-LDH/RGO/g-C_3N_4 hybrid as a Z-scheme photocatalyst for efficient and selective CO_2 reduction. *Appl. Catal. B* **255**, 117771 (2019).

13 Zhou, S. et al. Facile in situ synthesis of graphitic carbon nitride (g-C_3N_4)-N-TiO_2 heterojunction as an efficient photocatalyst for the selective photoreduction of CO_2 to CO. *Appl. Catal. B* **158**, 20-29 (2014).

14 Zhang, X. X. et al. Surface co-modification with highly-dispersed Mn & Cu oxides of g-C_3N_4 nanosheets for efficiently photocatalytic reduction of CO_2 to CO and CH_4. *Appl. Surf. Sci.* **492**, 125 (2019).

15 Liang, Y. J. Wu, X. Liu, X. Y. Li, C. H. & Liu. S. W. Recovering solar fuels from photocatalytic CO_2 reduction over W^{6+}-incorporated crystalline g-C_3N_4 nanorods by synergetic modulation of active centers. *Appl. Catal. B* **304**, 120978 (2022).

16 Chen, P. et al. Rare-earth single-atom La-N charge-transfer bridge on carbon nitride for highly efficient and selective photocatalytic CO_2 reduction. *ACS Nano* **14**, 15841-15852 (2020).

17 Ma, B. et al. Efficient visible-light-driven CO_2 reduction by a cobalt molecular catalyst covalently linked to mesoporous carbon nitride. *J. Am. Chem. Soc.* **142**, 6188-6195 (2020).

18 Jiang, J. et al. Van der waals heterostructures by single cobalt sites-anchored graphene and g-C_3N_4 nanosheets for photocatalytic syngas production with tunable CO/H_2 ratio. *Appl. Catal. B* **295**, 120261 (2021).

19 Tang, Q. J. Sun, Z. X. Deng, S. Wang, H. Q. & Wu, Z. B. Decorating g-C_3N_4 with alkalinized Ti_3C_2 MXene for promoted photocatalytic CO_2 reduction performance. *J. Colloid Interface Sci.* **564**, 406-417 (2020).

20 Jing, H. et al. Metallic MoO_2-modified graphitic carbon nitride boosting photocatalytic CO_2 reduction via schottky junction. *Sol. RRL*. **4**, 1900416 (2019).

21 Sun, Z. M. et al. g-C_3N_4 foam/Cu_2O QDs with excellent CO_2 adsorption and synergistic catalytic effect for photocatalytic CO_2 reduction. *Environ. Int.* **130**, 104898 (2019).

22 Li, Y. Li, B. H. Zhang, D. N. Cheng, L. & Xiang, Q. J. Crystalline carbon nitride supported copper single atoms for photocatalytic CO_2 reduction with nearly 100% CO selectivity. *ACS Nano* **14**, 10552 (2020).

23 Yang, Y. et al. In situ no-slot joint integration of half-metallic $C(CN)_3$ cocatalyst into g-C_3N_4 scaffold: an absolute metal-free in-plane heterosystem for efficient and selective photoconversion of CO_2 into CO. *Appl. Catal. B* **264**, 118470 (2020).

24 Zhang, X. D. Yan, J. Zheng, F. Y. Zhao, J. & Lee, L. Y. S. Designing charge transfer route at the interface between WP nanoparticle and g-C_3N_4 for highly enhanced photocatalytic CO_2 reduction reaction. *Appl. Catal. B* **286**, 119879 (2021).

25 Jiang, L. S. Wang, K. Wu, X. Y. & Zhang, G. K. Highly enhanced full solar spectrum-driven photocatalytic CO_2 reduction performance in $Cu_{2-x}S$/g-C_3N_4

composite: efficient charge transfer and mechanism insight. *Sol. RRL* **5**, 2000326 (2020).
